# Supplementary material for: HDAC4 regulates hASC osteogenesis and bone regeneration by mediating SMAD4 via histone acetylation
Source: Stem Cell Reports. 2026 Jun 11;21(7):102955. doi: 10.1016/j.stemcr.2026.102955 (PMC13385728; doi:10.1016/j.stemcr.2026.102955)
Supplement: Document S1. Figures S1–S6 and Tables S1–S4 [file mmc1.pdf]

**Stem Cell Reports, Volume *21***

## Supplemental Information

# HDAC4 regulates hASC osteogenesis and bone regeneration by mediating SMAD4 via histone acetylation

**Liyuan Yu, Kai Xia, Yijue Wang, Zhiai Hu, Jun Liu, Shujuan Zou, and Jianwei Chen**

**Supplemental Information**

**Document S1. Figures S1–S6**

**Table S1. Synthesized sequences of siRNA.**

**Table S2. Primer sequences of RT-qPCR.**

**Table S3. Primer sequences of CUT&Tag-qPCR**

**Table S4. Primer sequences of Human-specific Alu**

**1. Figure S1 CUT&Tag sequencing revealed HDAC4 inhibition enhances H3 acetylation modification during hASC osteogenic differentiation.**

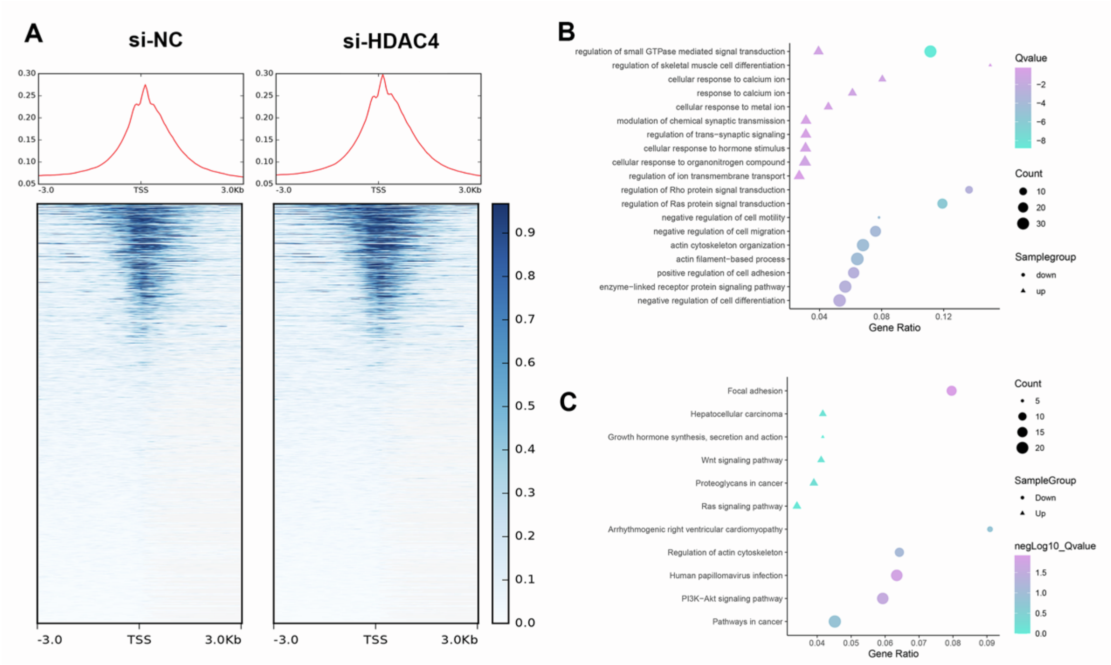


(A) The line graph shows the peaks identified by CUT&Tag enrichment around the transcription start site (TSS). The x-axis represents the distance from TSS, while the y-axis represents the degree of peak enrichment. The clustered heatmap reveals enriched peaks near the TSS, with blue indicating the level of enrichment. (B) GO analysis of differential peaks. (C) KEGG analysis of differential peaks.

**2. Figure S2 Knockdown of HDAC4 promotes cranial bone defect repair.**

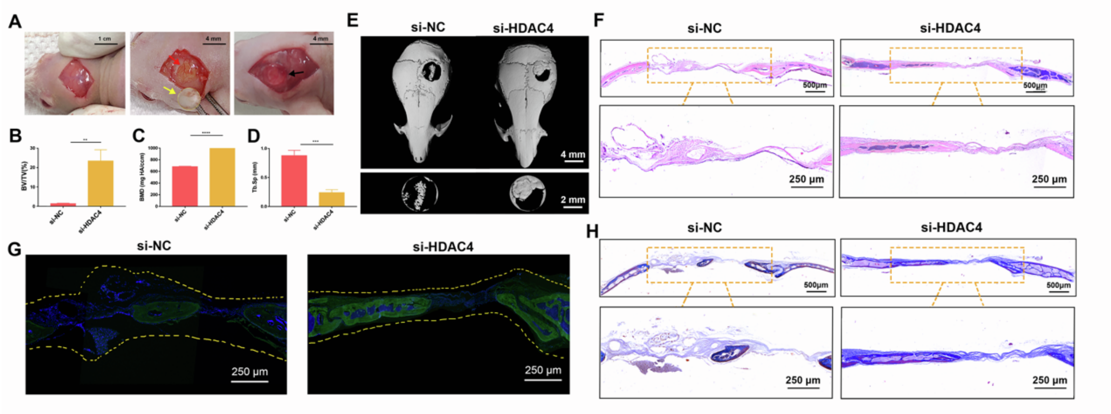


(A) Surgical images show the establishment of a critical-sized calvarial defect model in mice and the implantation of hydrogel microspheres. Red arrow indicates the defect area; black arrow indicates the implanted microspheres; yellow arrow indicates the explanted calvarial tissue. Scale bars: 1 cm (overview), 4 mm (magnified view). (B, C, D) Quantitative micro-CT analysis of bone repair parameters: bone volume fraction (BV/TV), bone mineral density (BMD) and trabecular spacing (Tb.Sp) in si-HDAC4 versus si-NC groups. Data are mean ± SD (n = 5 mice per group from three independent experiments). Statistical significance was determined by an unpaired two-tailed t-test. **p < 0.01, ***p < 0.001, ****p < 0.0001. (E) The three-dimensional reconstruction of the cranial bone of si-HDAC4 and si-NC groups. Lower images show magnified views of the defect areas. Scale bars: 4 mm (overview), 2 mm (magnified view). (F) Hematoxylin and eosin staining. Scale bars: 500 µm (overview), 250 µm (magnified view). (G)The immunofluorescence staining of the osteogenic marker COL1A1 (green); nuclei were counterstained with DAPI (blue). Scale bar: 250 µm. (H) Masson staining with blue staining indicating collagenous matrix. Scale bars: 500 µm (overview), 250 µm (magnified view).

**3. Figure S3 High-resolution histological analysis of mandibular defect repair delineating the interface between pre-existing host bone and new bone formation.**

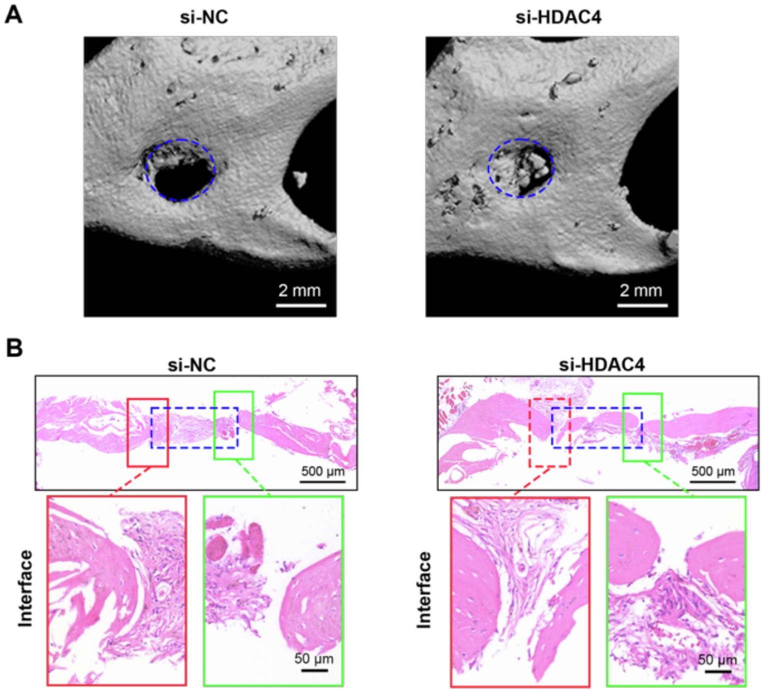


(A) Micro-CT 3D reconstruction of the mandibular defect region. The blue dashed circle indicates the central area of the original critical-sized defect. Scale bar: 2 mm. (B) Hematoxylin and eosin staining show the overall defect repair area. The blue dashed box outlines the central area of the original critical-sized defect. The red and green boxes indicate high-magnification views of the interface: the pre-existing host bone at the defect margin, characterized by dense, lamellar architecture; and the newly formed bone within the defect, displaying immature, porous woven bone. Scale bars: 500 μm (overview), 50 μm (insets).

**4. Figure S4 SMAD4 knockdown inhibits osteogenic differentiation of hASCs.**

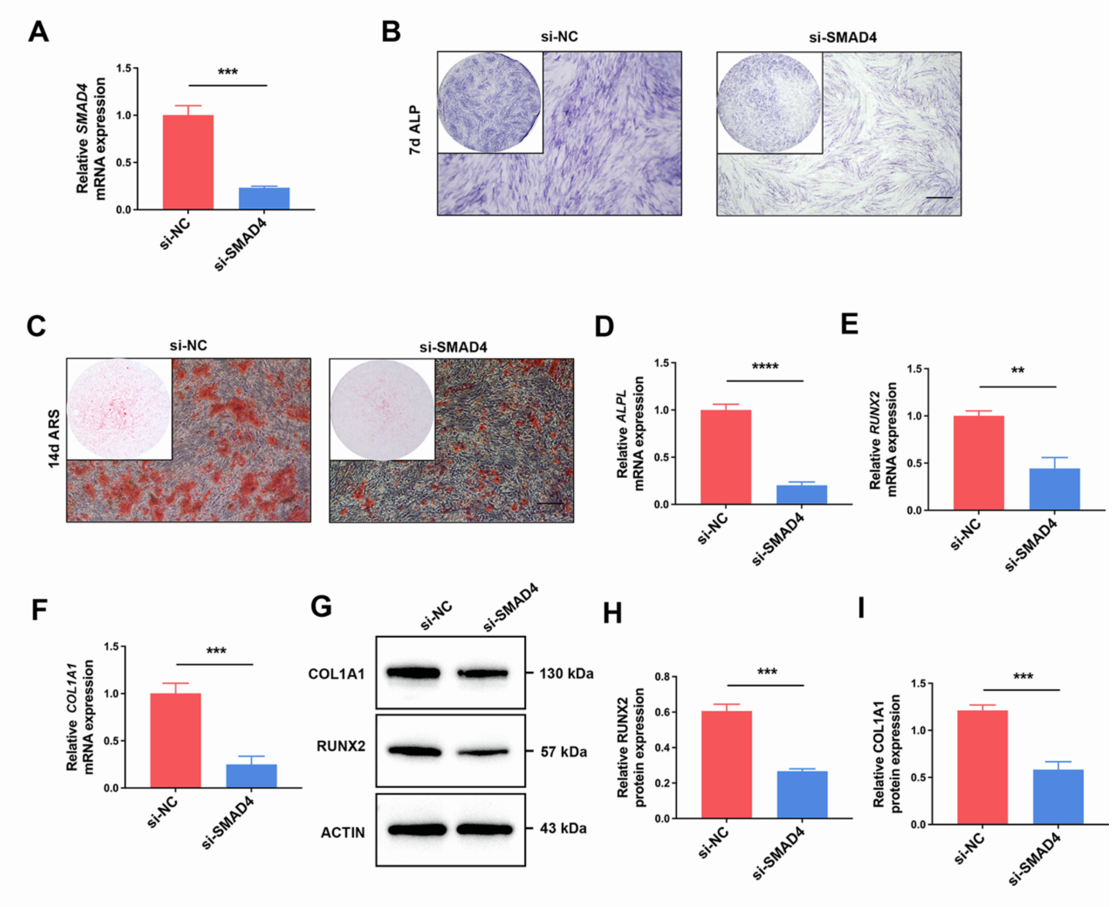


(A) RT qPCR analysis of *SMAD4* mRNA expression in hASCs following transfection with si-SMAD4 or si-NC. Data are mean ± SD (n = 3 independent experiments). Statistical significance was determined by an unpaired two-tailed t-test. ***p < 0.001. (B, C) ALP staining at day 7 and ARS staining at day 14 under osteogenic induction in si-SMAD4 versus si-NC. Scale bars: 200 µm. (D, E, F) RT-qPCR analysis of the osteogenic marker genes *ALPL*, *RUNX2*, and *COL1A* in si-SMAD4 versus si-NC. Data are mean ± SD (n = 3 independent experiments). Statistical significance was determined by an unpaired two-tailed t-test. **p < 0.01, ***p < 0.001, ****p < 0.0001. (G) Western blot detects the protein expression of RUNX2 and COL1A1 in si-SMAD4 versus si-NC. (H, I) Quantification of the protein levels of RUNX2 and COL1A1. Data are mean ± SD (n = 3 independent experiments). Statistical significance was determined by an unpaired two-tailed t-test. ***p < 0.001.

**5. Figure S5 Composition Schematic Diagram of GelMA-F127 Hydrogel Loaded with Tasquinimod.**

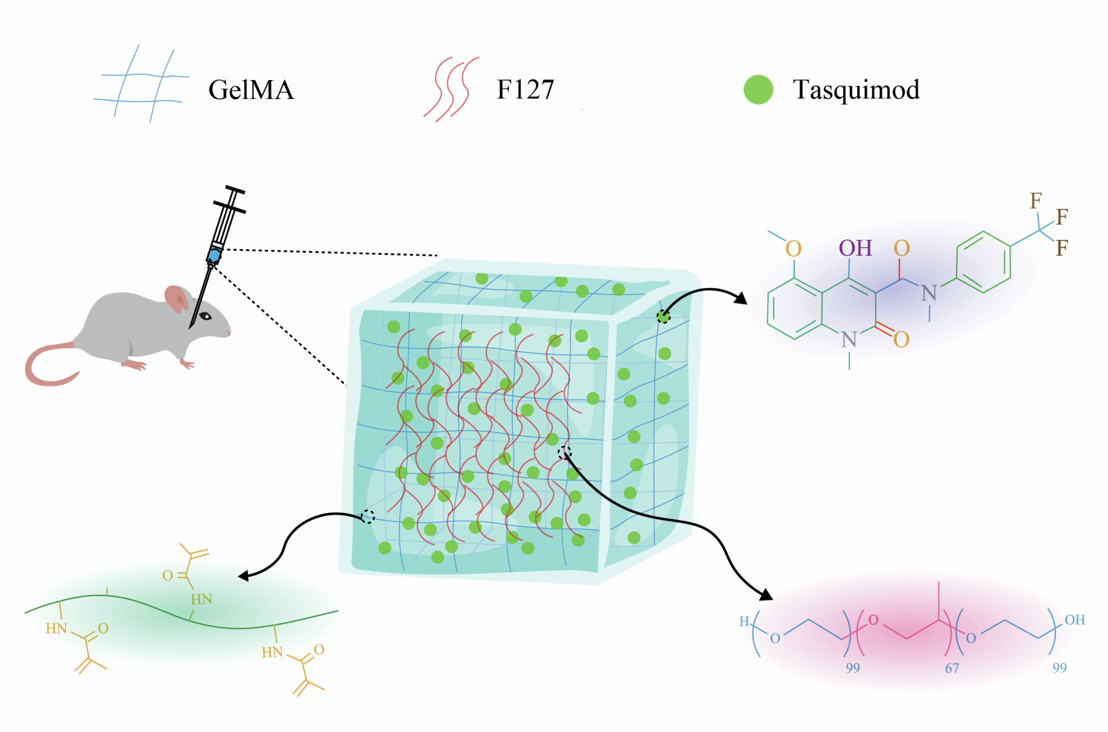

6. **Figure S6 *In vitro* Tasqunimod release kinetics and *in vivo* engraftment verification of hASCs.**

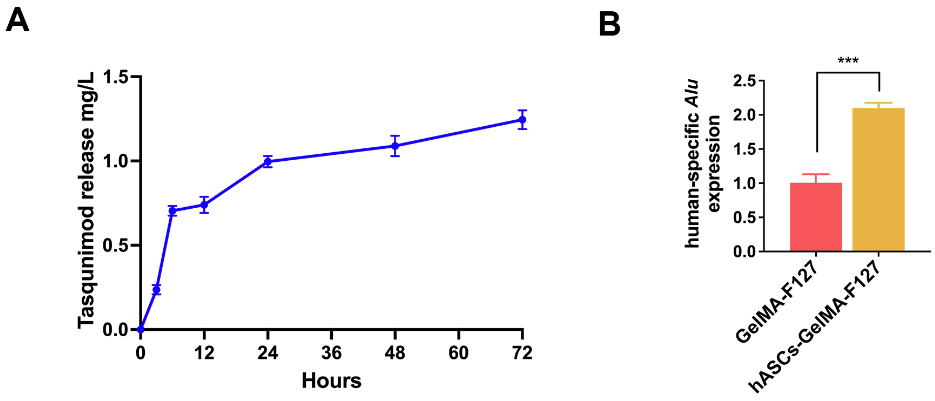


(A) *In vitro* release profile of Tasquinimod (Tasq) from GelMA-F127 hydrogel over 72 hours in PBS (pH 7.4, 37°C). Data are presented as mean ± SD (n = 3 independent experiments). The profile shows an initial rapid release phase followed by sustained release. (B) In vivo detection of transplanted human cells. Genomic DNA from mandibular defect sites 8 weeks post-implantation was analyzed by qPCR for human-specific *Alu* sequences. A significant signal was detected in the hASCs-GelMA-F127 group compared to the cell-free control (GelMA-F127), confirming donor cell engraftment. Data are presented as mean ± SD (n = 3 independent experiments). Statistical significance was determined by an unpaired two-tailed Student’s t-test (***p < 0.001).

**Table S1. Synthesized sequences of siRNA.**

| Genes | Sequences (5'-3') |
|---|---|
| HDAC4 | GCAGCAGCAUCAGCAGUUUTT |
| | AAACUGCUGAUGGUGCUGCTT |
| SMDA4 | GGUGGAGAGAGUGAAACAUTT |
| | AUGUUUCACUCUCUCCACCTT |
| NC | UUCUCCGAACGUGUCACGUTT |
| | ACGUGACACGUUCGGAGAATT |

**Table S2. Primer sequences of RT-qPCR.**

| Genes | Primer sequences (5'-3') |
|---|---|
| **GAPDH** | Forward: CTTTGGTATCGTGGAAGGACTC<br>Reverse: GTAGAGGCAGGGATGATGTTCT |
| **SMAD4** | Forward: ACTCCTACAACCTAGCCACCTTCTC<br>Reverse: GTTCTGTGTTGAGGACCTGCGTAG |
| **BGLAP** | Forward: CTACCTGTATCAATGGCTGGG<br>Reverse: GGATTGAGCTCACACACCT |
| **HDAC4** | Forward: GCCAAAGATGACTTCCCTCTTA<br>Reverse: TTTCGGCCACTTTCTGCTTTAG |
| **COL1A1** | Forward: CCAGTGTGGCCCAGAAGAAC<br>Reverse: TGGCCGCCATACTCGAACT |
| **MAP2K2** | Forward:CCTACATGGCTCCACCTCCTAAG<br>Reverse:ACCTCGGACCGCTTGATGAAG |
| **AKT2** | Forward:TGCGGAAGGAAGTCATCATTGC<br>Reverse:TCGTGGGTCTGGAAGGCATAC |
| **HDAC7** | Forward:ACCTCAATGCCATCCGCTCTC<br>Reverse:CACTGCCTCCACTTCTTCTTTGTC |
| **HDAC5** | Forward:ACGCTAGATGAGATCCAGACAGTG<br>Reverse:CCACAAGGCAGCACAGCATAC |

**Table S3. Primer sequences of CUT&Tag-qPCR**

| Genes | Primer sequences (5’-3’) |
| --- | --- |
| **SMAD4** | Forward: CACGCACAGTCCTTGGTTGG<br>Reverse: CTGATGCTGCTGCTGGATGAC |

**Table S4. Primer sequences of Human-specific Alu**

| Genes | Primer sequences (5’-3’) |
| --- | --- |
| | Forward: TCGCCCAGGCTGGAGTGCA<br>Reverse: CACCTGTAATCCCAGCACTTT |
