# Supplementary material for: HDAC4 regulates hASC osteogenesis and bone regeneration by mediating SMAD4 via histone acetylation
Source: Stem Cell Reports. 2026 Jun 11;21(7):102955. doi: 10.1016/j.stemcr.2026.102955 (PMC13385728; doi:10.1016/j.stemcr.2026.102955)
Supplement: Document S2. Article plus supplemental information [file mmc2.pdf]

STEM CELL REPORTS
Article
OPEN ACCESS

HDAC4 regulates hASC osteogenesis and bone regeneration by mediating SMAD4 via histone acetylation

Liyuan Yu,[1,2,4] Kai Xia,[1,3,4] Yijue Wang,[1] Zhiai Hu,[1] Jun Liu,[1] Shujuan Zou,[1] and Jianwei Chen[1,5,*]
[1]State Key Laboratory of Oral Diseases & National Clinical Research Center for Oral Diseases, Department of Orthodontics, West China Hospital of Stomatology, Sichuan University, Chengdu 610106, P.R. China
[2]Centre of Craniofacial Orthodontics, Department of Oral and Maxillofacial Surgery, Shanghai Ninth People's Hospital, Shanghai Jiao Tong University School of Medicine, College of Stomatology, Shanghai Jiao Tong University, National Center for Stomatology, National Clinical Research Center for Oral Diseases, Shanghai Key Laboratory of Stomatology, Shanghai Research Institute of Stomatology, Shanghai 200011, P.R. China
[3]Center Laboratory of Dental Biomaterials and Tissue Regeneration, Shanghai Xuhui District Stomatological Hospital, Shanghai 200032, P.R. China
[4]These authors contributed equally
[5]Lead contact
*Correspondence: chenjianwei2017@vip.163.com
https://doi.org/10.1016/j.stemcr.2026.102955

SUMMARY

Craniofacial large bone defects possess limited self-repair capacity. This study identifies histone deacetylase 4 (HDAC4) as a key epigenetic repressor of osteogenesis in human adipose-derived stem cells. Inhibiting HDAC4, either genetically or pharmacologically with tasquinimod, enhanced histone acetylation and activates SMAD4 expression, thereby promoting bone formation. A sustained-release hydrogel delivering tasquinimod was developed and demonstrated to significantly enhance bone regeneration in critical-sized cranial and mandibular defects in mice. The findings reveal a promising localized epigenetic strategy for repairing craniofacial bone defects.

INTRODUCTION

Critical-size defects (CSDs) in the craniofacial region represent a significant clinical challenge in oral and maxillofacial surgery, primarily caused by trauma, tumor resection, or infection. These defects are frequently associated with substantial functional impairment and facial disfigurement, severely impacting patients' quality of life. Due to the limited self-healing capacity in this region, current clinical repair methods, including autologous bone grafts, allogeneic bone grafts, and bone substitutes, exhibit inherent limitations (Wu et al., 2022). Autologous grafts are constrained by donor site morbidity and limited availability. Allogeneic grafts, while offering wider availability, carry risks of immune rejection and infection. Bone substitutes often lack sufficient osteoinductive properties (Yu et al., 2023). Consequently, there is an urgent need to develop novel, safe, and highly effective strategies for bone defect repair.

In recent years, bone tissue engineering has garnered significant attention as a cutting-edge alternative to traditional transplantation methods. Bone tissue engineering, using scaffolds loaded with osteogenic factors or cells to simulate osteogenesis, has recently emerged as a promising alternative for repairing CSDs (Xu et al., 2022). Human adipose-derived stem cells (hASCs) are derived from abundant adipose tissue and have the advantages of minimal invasiveness and low immunogenicity, which make them ideal for bone tissue engineering (Li and Guo, 2018).

The regulatory mechanisms governing osteogenic differentiation are complex. Recent research has increasingly focused on the role of epigenetic modifications in this process. Notably, histone acetylation, a critical mechanism for modulating chromatin architecture, regulates cellular fate by controlling gene transcription activity (Gomathi et al., 2021). Histone acetyltransferases can loosen chromatin structure, promoting gene activation. Conversely, histone deacetylases (HDACs) regulate deacetylation, resulting in a more densely coiled chromatin structure and thereby inhibiting gene transcription. HDAC4, a class IIa HDAC, is a zinc-dependent enzyme that regulates histone deacetylation and plays a pivotal role in osteogenesis and chondrogenesis (Wang et al., 2014). Previous studies have demonstrated that HDAC4 can bind to and suppress the expression of the osteogenic transcription factors RUNX2 (Guan et al., 2012) and ATF4 (Chen et al., 2023), thereby impeding osteogenic differentiation. Its activity is also involved in parathyroid hormone (PTH)-mediated bone metabolism regulation (Blixt et al., 2017).

Tasquinimod (Tasq) is a small-molecule selective inhibitor of HDAC4 that binds to its Zn^{2+} domain, locking it in an inactive conformation, thereby promoting histone acetylation and enhancing gene expression. Originally developed as an anti-prostate cancer agent (Isaacs et al., 2013), Tasq has been recently found to modulate the bone microenvironment and osteogenic signaling pathways, suggesting its potential therapeutic value for bone regeneration (Magnusson et al., 2016). Our previous research confirmed that HDAC4 expression is downregulated during the osteogenic differentiation of hASCs, and knocking down HDAC4 significantly enhances their osteogenic potential (Yu et al., 2022). However, it remains unclear whether

© 2026 The Authors.
Published by Elsevier Inc. on behalf of International Society for Stem Cell Research.
This is an open access article under the CC BY license (http://creativecommons.org/licenses/by/4.0/).

HDAC4 mediates the expression of osteogenic genes (e.g., *SMAD4*) through histone acetylation modifications, and whether this regulatory mechanism can be mimicked by Tasq for bone defect therapy remains to be systematically investigated.

Therefore, this study is based on the following core hypothesis: HDAC4 suppresses osteogenic differentiation of hASCs through histone acetylation, and its inhibition enhances the expression of osteogenic key factors such as SMAD4, thereby promoting bone formation. We employed CUT&Tag sequencing, combined with bioinformatics approaches, to delineate the impact of HDAC4 knockdown on the histone acetylation landscape and osteogenic phenotype of hASCs. Simultaneously, we evaluated the role of Tasq in osteogenesis induction both *in vitro* and *in vivo* and developed a novel Tasq-loaded GelMA-F127 hydrogel for localized bone defect repair. This study aims to elucidate the epigenetic regulatory mechanisms of HDAC4 and its targeting strategies, providing a theoretical basis and translational technological pathways for the treatment of craniofacial bone defects.

RESULTS

HDAC4 inhibition enhances the expression of the osteogenic regulator SMAD4 in hASCs via promoting histone acetylation

To elucidate the epigenetic regulatory role of HDAC4 in the osteogenic differentiation of hASCs, we first knocked down HDAC4 and analyzed its effect on histone acetylation by using CUT&Tag technology. Knockdown of HDAC4 led to a pronounced increase in AcH3 levels at the transcription start site (TSS ±3 kb), with a genome-wide normal distribution pattern (Figure S1A). Cluster analysis (Figure 1A) and Venn diagrams (Figure 1B) revealed that the si-HDAC4 group had significantly increased osteogenesis-related acetylation peaks, with 25,883 peaks uniquely present in this group. Further, Gene Ontology (GO) and Kyoto Encyclopedia of Genes and Genomes (KEGG) analyses (Figures S1B and S1C) revealed that these upregulated acetylation regions were enriched in functions such as skeletal muscle cell differentiation, cell adhesion, ion transport, and calcium ion response and showed significant associations with osteogenic signaling pathways including Wnt, Ras, and PI3K-AKT. These findings suggest that HDAC4 negatively regulates osteogenic gene expression.

Immunofluorescence (IF) staining confirmed enhanced AcH3 expression in the si-HDAC4 group (Figures 1C and 1D). Integrative Genomics Viewer (IGV) analysis (Figure 1E) further demonstrated increased acetylation levels at key osteogenic genes such as *AKT2*, *MAP2K2*, *RUNX2*, and *SMAD4* in the si-HDAC4 group. Motif analysis indicated that HDAC4-regulated acetylation binding sites strongly correlated with transcription factors critical for osteogenesis, such as RUNX2 and SMAD4 (Figure 1F). Consistent with the elevated histone acetylation, quantitative reverse-transcription PCR (RT-qPCR) analysis showed that the mRNA expression levels of *AKT2*, *MAP2K2, RUNX2*, and *SMAD4* were all significantly upregulated following HDAC4 knockdown (Figures 1G–1J), indicating that the enhanced acetylation at promoter/enhancer regions functionally promotes gene expression. To investigate potential compensatory effects from other class IIa HDACs, we examined HDAC5 and HDAC7 expression after HDAC4 knockdown. Western blot (Figure 2A) and RT-qPCR (Figures 2B and 2C) showed that neither their mRNA nor protein levels were upregulated upon HDAC4 knockdown, indicating that HDAC4 regulates osteogenic differentiation independently.

To verify whether SMAD4 is a direct target of HDAC4, we first assessed histone acetylation at its promoter region using CUT&Tag-qPCR. The results revealed a significant increase in AcH3 enrichment at the *SMAD4* promoter upon HDAC4 knockdown (Figure 2D), indicating enhanced histone acetylation. Concordantly, western blot (Figure 2E) confirmed a marked upregulation of SMAD4 levels. To directly test HDAC4 binding, we performed chromatin immunoprecipitation (ChIP), which demonstrated that HDAC4 specifically associates with the *SMAD4* promoter region (Figures 2F–2H). Collectively, these results suggest that HDAC4 directly binds to the *SMAD4* promoter and represses its expression by deacetylating histone H3. During osteogenic differentiation of hASCs, this repression is alleviated upon HDAC4 knockdown, leading to enhanced SMAD4 expression and elevated osteogenic potential.

Knockdown of HDAC4 enhances the osteoregenerative capacity of hASCs and promotes cranial bone defect repair

After confirming the regulatory role of HDAC4 in osteogenic phenotype, we further validated its function using a CSD model in nude mice. si-HDAC4-hASCs were loaded onto GelMA hydrogel and transplanted into the defect area (Figure S2A). Micro-CT three-dimensional (3D) reconstruction (Figure S2E) revealed more robust new bone formation and significantly improved defect filling in the si-HDAC4 group. Quantitative bone analysis (Figures S2B–S2D) showed marked increases in bone volume fraction (BV/TV) and bone mineral density (BMD), alongside reduced trabecular spacing (Tb.Sp), indicating enhanced bone quality. Hematoxylin and eosin (H&E) and Masson staining further confirmed that the si-HDAC4 group exhibited denser bone structure and more uniform collagen distribution, indicative of mature bone formation (Figures S2F and S2H). IF staining for COL1A1

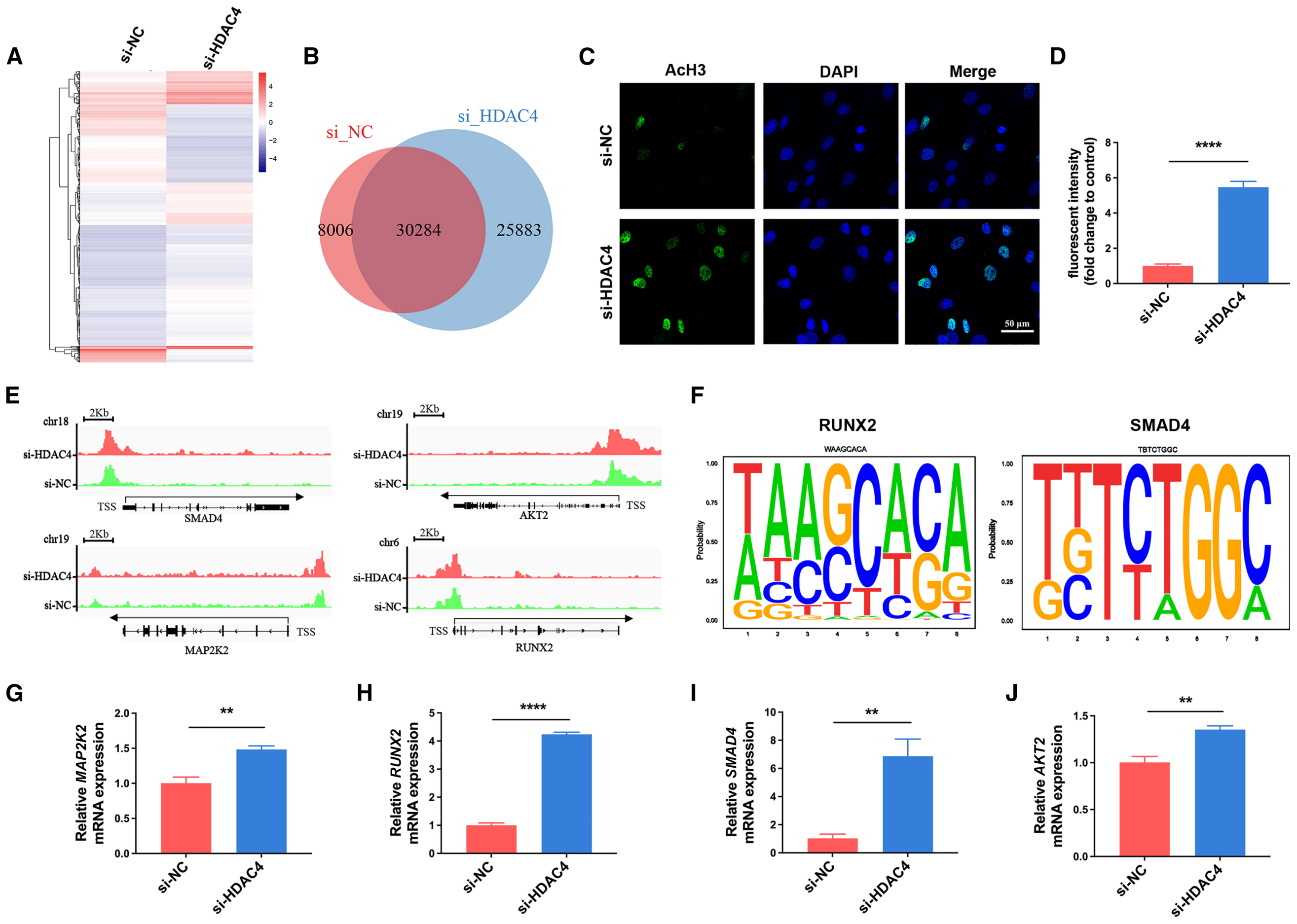

Figure 1. HDAC4 regulates histones acetylation modification during hASC osteogenesis

(A) Hierarchical clustering analysis of acetylated histone H3 (AcH3) peaks in the si-HDAC4 versus si-NC groups during the osteogenic differentiation of hASCs. Red indicates upregulated expression peaks, while blue indicates downregulated expression peaks.
(B) Venn diagrams showing the number of unique and shared AcH3 peaks between the si-HDAC4 and si-NC groups.
(C) Immunofluorescence staining of AcH3 (green) and DAPI (blue) after osteogenic differentiation for 7 days. Scale bars: 50 μm.
(D) Quantification of AcH3 fluorescence intensity. Data are mean ± SD (n = 3 independent experiments). Statistical significance was determined by an unpaired two-tailed t test. ****$p < 0.0001$.
(E) IGV visualization analysis showing AcH3 enrichment at the sites of osteogenic genes (*AKT2*, *MAP2K2*, *RUNX2*, and *SMAD4*) in the si-HDAC4 and si-NC groups.
(F) The motif with the highest enrichment in the AcH3 peak region regulated by HDAC4, including the binding sites of RUNX2 and SMAD4.
(G–J) RT-qPCR verified the mRNA expression of *MAP2K2*, *RUNX2*, *AKT2*, and *SMAD4* in si-HDAC4 versus si-NC groups.

also indicated enhanced new bone synthesis activity (Figure S2G). These findings suggest that knockdown of HDAC4 in hASCs effectively promotes calvarial defect regeneration *in vivo*, demonstrating their potential for application in bone tissue engineering.

Knockdown of HDAC4 also promotes mandibular bone defect repair

To determine whether the above effect extends to mandibular defects, we created 2.3 mm CSD in nude mice and treated it with si-HDAC4-transfected hASCs (Figure 3A). Micro-CT reconstruction (Figure 3B) revealed more complete defect filling and significantly increased bone volume in the si-HDAC4 group. The defect margin, indicated by a blue dashed line in the micro-CT image (Figure S3A), appeared smooth and dense, while the new bone inside showed lower density and a porous structure. Correspondingly, H&E staining (Figure S3B) confirmed that the new bone, outlined by a dashed boundary, was primarily located inside the defect area, displaying a discontinuous, porous, and mesh-like trabecular structure. Quantitative analysis showed significant increases in BV/TV, BMD, and trabecular thickness (Tb.Th), along with a reduction in Tb.Sp (Figure 3C). Consistent results

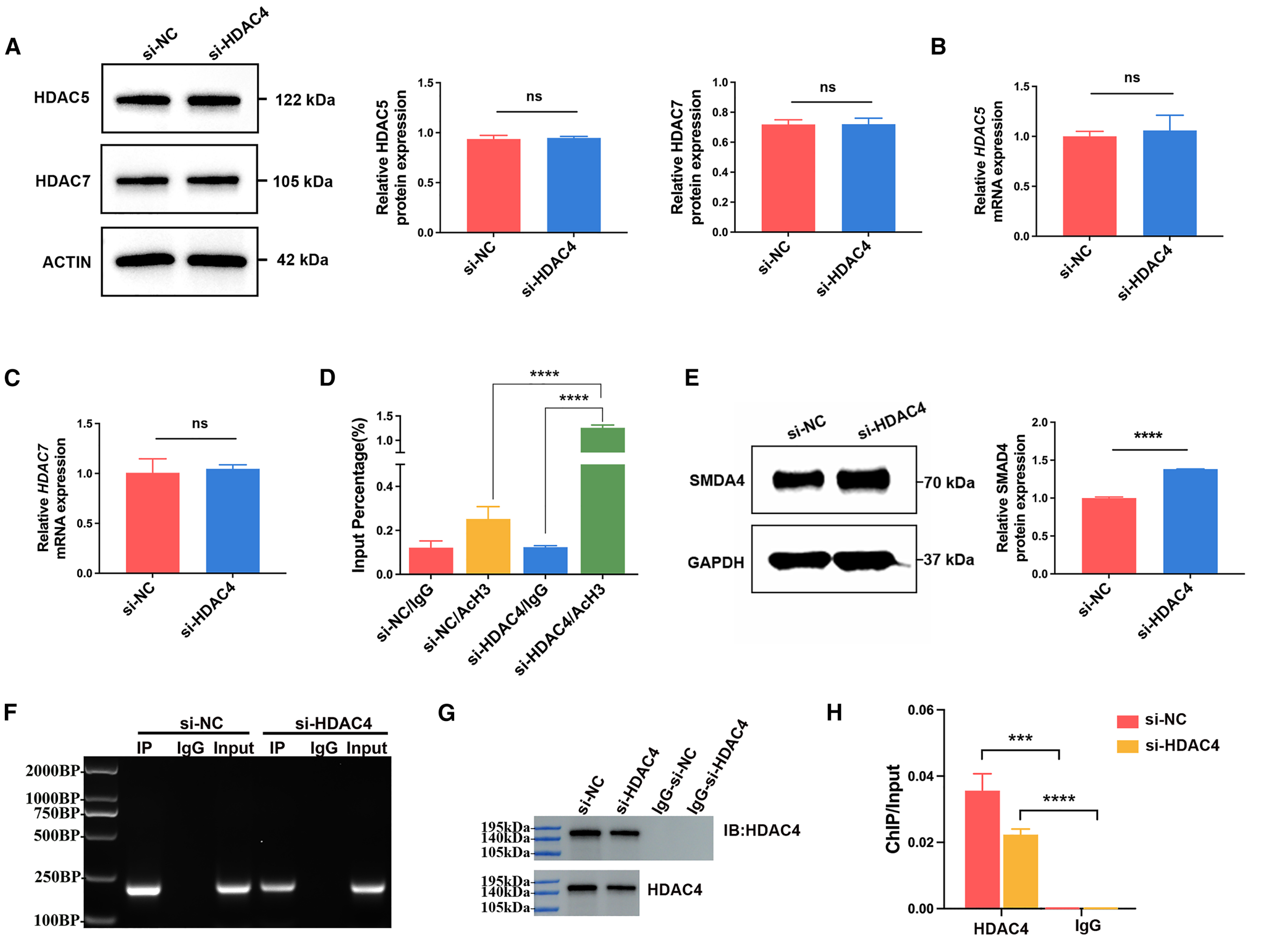

Figure 2. HDAC4 directly binds to the *SMAD4* promoter region

(A) Western blot and quantitative analysis detected the expression levels of HDAC5 and HDAC7 in si-HDAC4 versus si-NC groups. Data are mean ± SD (n = 3 independent experiments). Statistical significance was determined by an unpaired two-tailed t test. ns, not significant.

(B and C) RT-qPCR detected the mRNA expression of *HDAC5* and *HDAC7* in si-HDAC4 versus si-NC groups. Data are mean ± SD (n = 3 independent experiments). Statistical significance was determined by an unpaired two-tailed t test. ns, not significant.

(D) CUT&Tag-qPCR confirmed histone H3 acetylation (AcH3) enrichment at the *SMAD4* promoter region in si-HDAC4 versus si-NC groups. Data are mean ± SD (n = 3 independent experiments). Statistical significance was determined by an unpaired two-tailed t test. ****$p < 0.0001$.

(E) Western blot and quantitative analysis detected SMAD4 protein levels in si-HDAC4 and si-NC groups. Data are mean ± SD (n = 3 independent experiments). Statistical significance was determined by an unpaired two-tailed t test. ****$p < 0.0001$.

(F) Agarose gel electrophoresis of PCR products from the ChIP assay, showing amplified DNA fragments from the *SMAD4* promoter region in si-NC and si-HDAC4 groups. "Input" represents 1% of the starting chromatin prior to immunoprecipitation. IP represents immunoprecipitation.

(G) Western blot validation of the HDAC4 antibody specificity for ChIP. Cell lysates from si-NC- and si-HDAC4-treated cells were subjected to IP with either anti-HDAC4 antibody or control IgG, followed by immunoblotting (IB) with an anti-HDAC4 antibody.

(H) ChIP-qPCR quantification of HDAC4 enrichment at the *SMAD4* promoter in si-HDAC4 versus IgG control groups. Data are presented as the percentage of "input" and are mean ± SD (n = 3 independent experiments). Statistical significance was determined by an unpaired two-tailed t test. ***$p < 0.001$; ****$p < 0.0001$.

were observed in histological staining (Figures 3D and 3E) and IF analysis (Figures 3F and 3G), demonstrating increased expression of the osteogenic markers COL1A1 and ALP following si-HDAC4 treatment. These findings indicate that HDAC4 knockdown significantly enhances bone regeneration in both calvarial and mandibular defects, broadening its potential for bone tissue engineering applications.

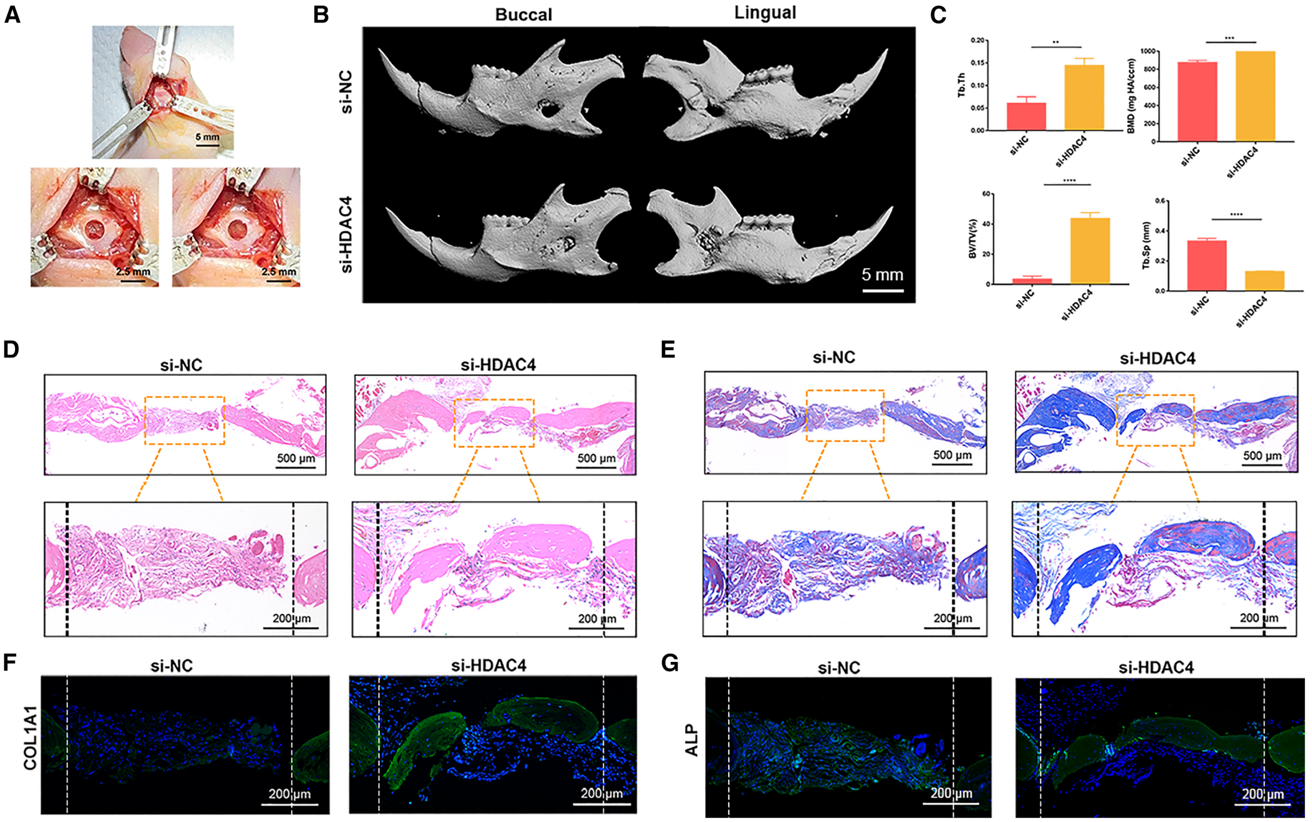

Figure 3. Knockdown of HDAC4 promotes mandibular bone defect repair
(A) Surgical images showing the establishment of the critical bone defect animal model and the implantation of hydrogel microspheres. Scale bars: 5 mm (overview) and 2.5 mm (magnified view).
(B) The three-dimensional reconstruction of the mandibles from the buccal and lingual views in si-HDAC4 versus si-NC groups. Scale bars: 5 mm.
(C) The measurement of the ratio of bone volume/total volume (BV/TV), bone mineral density (BMD), trabecular thickness (Tb.Th), and trabecular spacing (Tb.Sp) in si-HDAC4 versus si-NC groups. Data are mean ± SD ($n = 5$ mice per group from three independent experiments). Statistical significance was determined by an unpaired two-tailed t test. $^{**}p < 0.01$, $^{***}p < 0.001$, and $^{****}p < 0.0001$.
(D and E) Hematoxylin and eosin- and Masson-stained sections of the defect region. The dashed lines outline the central area of bone defect. Scale bars: 500 μm (overview) and 200 μm (magnified view).
(F and G) Immunofluorescence staining of the osteogenic marker COL1A1 (green); nuclei were counterstained with DAPI (blue). Dashed lines outline the central area of bone defect. Scale bars: 200 μm.

Tasq safely modulates the proliferation and viability of hASCs, with 1 μM identified as the optimal concentration

To explore an alternative approach for HDAC4 inhibition, we assessed the effects of Tasq on hASC proliferation and apoptosis across a concentration range (0, 1, 2.5, 5, and 10 μM), as previously reported (Isaacs et al., 2013; Brennen et al., 2016). Wound healing assay (Figure 4A) and CCK-8 analysis (Figure 4D) demonstrated that 0–2.5 μM Tasq significantly enhanced cell migration and proliferation, whereas concentrations ≥ 5 μM suppressed proliferation. Furthermore, apoptosis assays and live/dead staining further revealed that 1 μM Tasq effectively reduced early apoptosis without inducing noticeable cytotoxicity (Figures 4B and 4C). Collectively, these results confirmed that Tasq at 1 μM exhibits good biocompatibility with hASCs, providing a rational dose for subsequent osteogenic studies.

Tasq mimics HDAC4 knockdown to promote hASC osteogenic differentiation and enhance SMAD4 expression

We further investigated the effects of Tasq on hASC osteogenic differentiation. Alkaline phosphatase (ALP) staining (7 days) and Alizarin Red S (ARS) staining (14 days) revealed that the 1 μM group exhibited the strongest osteogenic potential, characterized by abundant mineralized nodules and enhanced staining intensity (Figure 5A). The

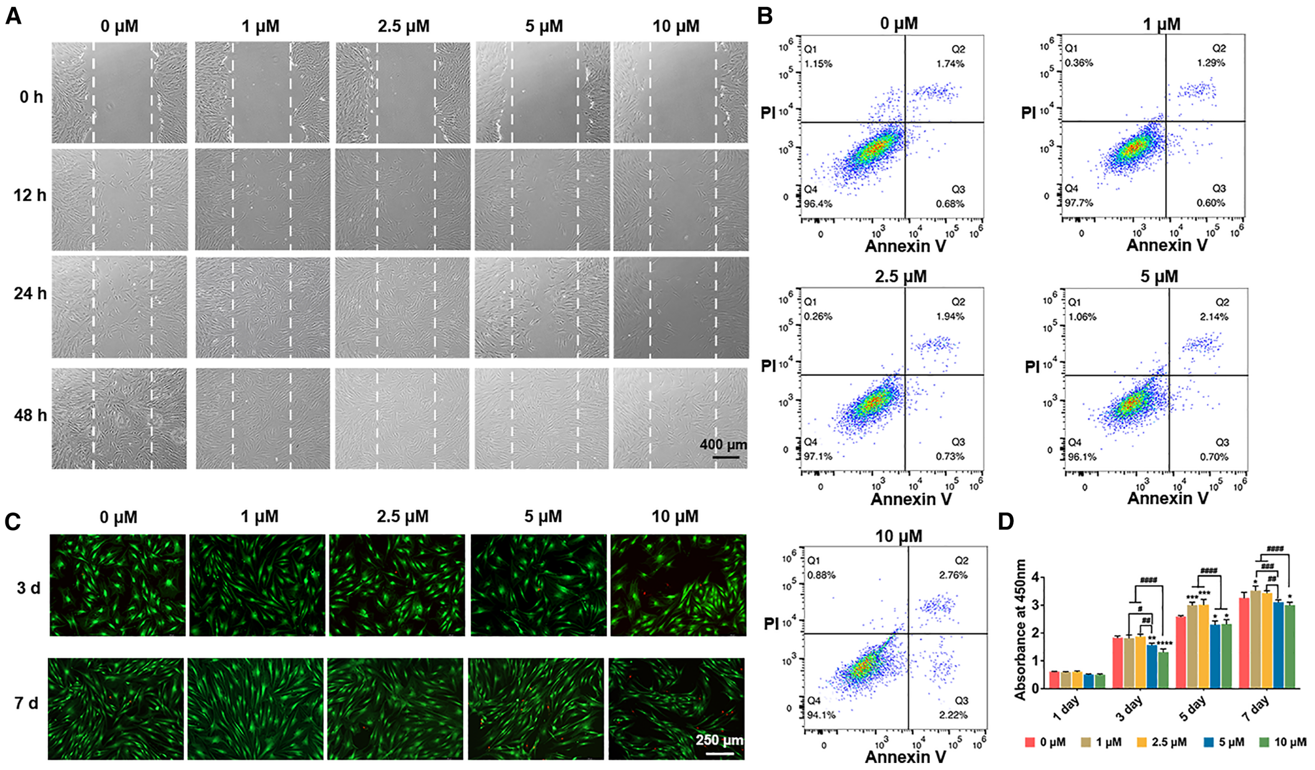

Figure 4. Effect of the HDAC4 inhibitor tasquinimod on the proliferation of hASCs

(A) Wound healing assay of hASCs treated with the indicated concentrations of tasquinimod (Tasq; 0, 1, 2.5, 5, and 10 μM) over 48 h. Scale bars: 400 μm.

(B) Apoptosis assays by flow cytometry following 72-h treatment with the indicated Tasq concentrations. Data are mean ± SD (n = 3 independent experiments).

(C) Live/dead cell staining (green: live cells; red: dead cells) of hASCs after 3 and 7 days of culture with the indicated Tasq concentrations. Scale bars: 250 μm.

(D) Cell proliferation assessed by CCK-8 assays at 1, 3, 5, and 7 days post-treatment with the indicated Tasq concentrations. Data are mean ± SD (n = 3 independent experiments).

Statistical significance was determined by two-way ANOVA followed by Sidak's test. */#p < 0.05; **/##p < 0.01; ***/###p < 0.001; ****/####p < 0.0001; * indicates the statistical difference from the 0 μM group at the same time.

expression of osteogenic marker genes (*BGLAP* and *COL1A1*) and proteins (ALP and OSX) peaked in the 1 μM group, while concentrations ≥ 5 μM exhibited inhibitory effects (Figures 5B–5D). Furthermore, IF staining demonstrated increased AcH3 expression (Figure 5E). RT-qPCR and western blot analyses confirmed that 1 μM Tasq treatment significantly upregulated SMAD4 expression, showing an approximately 7-fold increase at the mRNA level and a more modest 1.5-fold increase at the protein level (Figures 5F and 5G). This result indicated that Tasq activates SMAD4 expression, and the difference in multiplicative changes may reflect post-transcriptional regulatory mechanisms that modulate the translation efficiency or stability of SMAD4. Collectively, these findings demonstrate that 1 μM Tasq effectively mimics the effects of HDAC4 knockdown, activates SMAD4 expression, and enhances the osteogenic differentiation capacity of hASCs.

SMAD4 promotes osteogenic differentiation of hASCs

Knockdown of SMAD4 confirmed its essential role as a positive regulator of osteogenic differentiation in hASCs. The loss-of-function approach using si-SMAD4 successfully downregulated *SMAD4* mRNA expression (Figure S4A). Phenotypic analysis showed that SMAD4 knockdown significantly decreased the osteogenic capacity of hASCs, as indicated by reduced ALP staining at day 7 (Figure S4B) and decreased matrix mineralization (ARS staining) at day 14 (Figure S4C), compared with the si-NC control. At the molecular level, mRNA expression of the osteogenic markers *ALPL*, *RUNX2*, and *COL1A1* was markedly downregulated following SMAD4 knockdown (Figures S4D–S4F). This

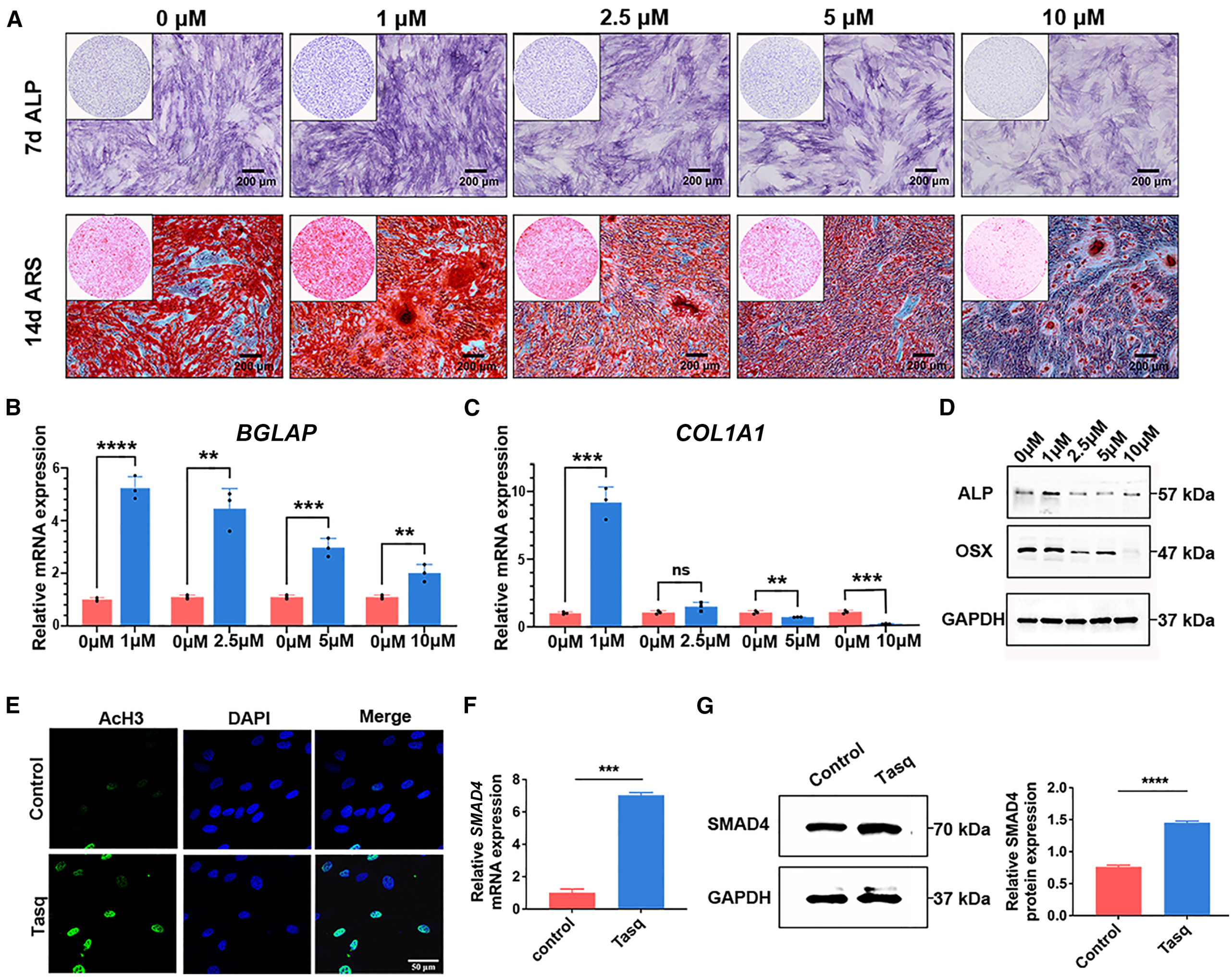

Figure 5. Tasquinimod promotes the osteogenic differentiation of hASCs and enhances H3 histone acetylation

(A) ALP staining (7 days) and ARS staining (14 days) of hASCs treated with the indicated concentrations of tasquinimod (Tasq; 0, 1, 2.5, 5, and 10 μM) during osteogenic induction.

(B and C) RT-qPCR analysis detected the mRNA expression levels of *BGLAP* and *COL1A1* in hASCs treated with the indicated Tasq concentrations for 7 days of osteogenic induction. Data are mean ± SD (n = 3 independent experiments). Statistical significance was determined by an unpaired two-tailed t test. **p < 0.01; ***p < 0.001; ****p < 0.0001; ns, not significant.

(D) Western blot detected the expression levels of ALP and OSX in hASCs treated with the indicated Tasq concentrations for 7 days of osteogenic induction.

(E) Immunofluorescence staining of acetylated histone H3 (AcH3) after 7 days of osteogenic induction with 0 μM (control group) or 1 μM Tasq (Tasq group). Blue fluorescence represents DAPI expression, while green fluorescence represents AcH3 expression. Scale bars: 50 μm.

(F and G) RT-qPCR analysis of *SMAD4* mRNA expression and western blot with quantification of SMAD4 in hASCs treated with 0 μM (control group) or 1 μM Tasq (Tasq group) for 7 days of osteogenic induction. Data are mean ± SD (n = 3 independent experiments). Statistical significance was determined by an unpaired two-tailed t test. ***p < 0.001; ****p < 0.0001.

inhibitory effect was further confirmed at the protein level, with substantially decreased RUNX2 and COL1A1 expression in the si-SMAD4 group (Figures S4G–S4I). Collectively, these findings demonstrate that SMAD4 serves as a critical promoter of osteogenesis in hASCs, as its knockdown suppresses osteogenic marker expression and impairs mineralized matrix formation and osteogenic differentiation.

GelMA-F127 loaded with Tasq exhibits structural stability, excellent properties, and biocompatibility

To achieve the localized application and sustained release of Tasq, we fabricated a Tasq-loaded GelMA-F127 (GelMA-F127-Tasq) composite hydrogel. The hydrogel structure is depicted in Figure S5. The stress-strain curve (Figure 6A) demonstrated enhanced compressive strength, while PBS

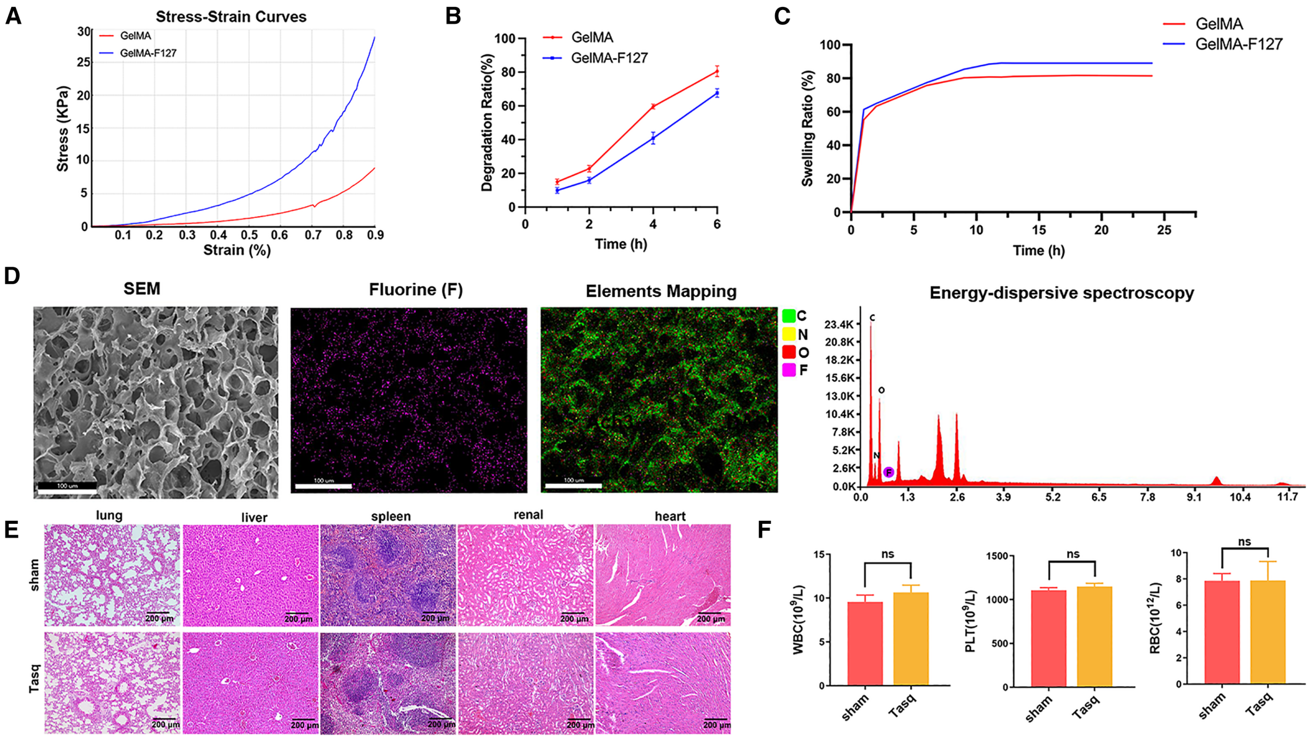

Figure 6. Physicochemical properties of GelMA-F127 and biosafety evaluation of GelMA-F127 loaded with tasquinimod

(A) The stress-strain curve of GelMA and GelMA-F127. Data are mean ± SD (n = 3 independent experiments).

(B) *In vitro* degradation profiles of GelMA and GelMA-F127 in PBS at 37°C. Data are mean ± SD (n = 3 independent experiments).

(C) Swelling assay of GelMA and GelMA-F127 in PBS at 37°C. Data are mean ± SD (n = 3 independent experiments).

(D) The surface microstructure of GelMA-F127-Tasq was observed using field-emission scanning electron microscopy, and elemental distribution was analyzed by X-ray energy-dispersive spectroscopy (EDS) elemental mapping. Green, carbon (C); yellow, nitrogen (N); red, oxygen (O); purple, fluorine (F).

(E) Hematoxylin and eosin staining of major organs (lung, liver, spleen, kidney, and heart) from the sham (no implant) and Tasq (GelMA-F127-Tasq implant) groups 8 weeks post-implantation.

(F) Hematological analysis of the red blood cell (RBC), white blood cell (WBC), and platelet (PLT) in peripheral blood 2 weeks post-implantation in sham versus Tasq groups. Data are mean ± SD (n = 5 mice per group from three independent experiments). Statistical significance was determined by an unpaired two-tailed t test. ns, not significant.

degradation assays demonstrated slower degradation kinetics (Figure 6B). The hydrogel exhibited swelling properties comparable to GelMA (Figure 6C). High-performance liquid chromatography revealed that Tasq was released from the hydrogel in a biphasic manner, with an initial burst phase followed by prolonged, sustained release over 72 h (Figure S6A). Furthermore, scanning electron microscopy (SEM) confirmed a continuous porous structure and successful Tasq incorporation (Figure 6D). Systemic toxicity evaluation indicated no toxicity to major organs (heart, liver, spleen, lungs, and kidneys) (Figure 6E), and hematological parameters showed no significant abnormalities (Figure 6F). Moreover, qPCR detection of human-specific *Alu* sequences confirmed the successful engraftment of hASCs delivered within the hydrogel, with a significant signal in the hASCs-GelMA-F127 group compared with that in the cell-free control (GelMA-F127) (Figure S6B). Taken together, these findings demonstrate that GelMA-F127 hydrogel serves as a promising carrier for Tasq delivery, combining robust mechanical properties with excellent biocompatibility.

GelMA-F127-Tasq effectively promotes mandibular bone defect repair, confirming the synergistic effect of the biomaterial and the drug

We applied the GelMA-F127-Tasq hydrogel to a mandibular bone defect model to validate its reparative efficacy *in vitro*. The experimental group (Tasq group) received GelMA-F127-Tasq at the defect site, while the control group received GelMA-F127 alone. 3D reconstruction (Figure 7A) showed that the Tasq group had significantly reduced defect areas and increased new bone formation compared with the control group. Bone-related parameters revealed higher BMD, BV/TV, and the trabecular number (Tb.N)in

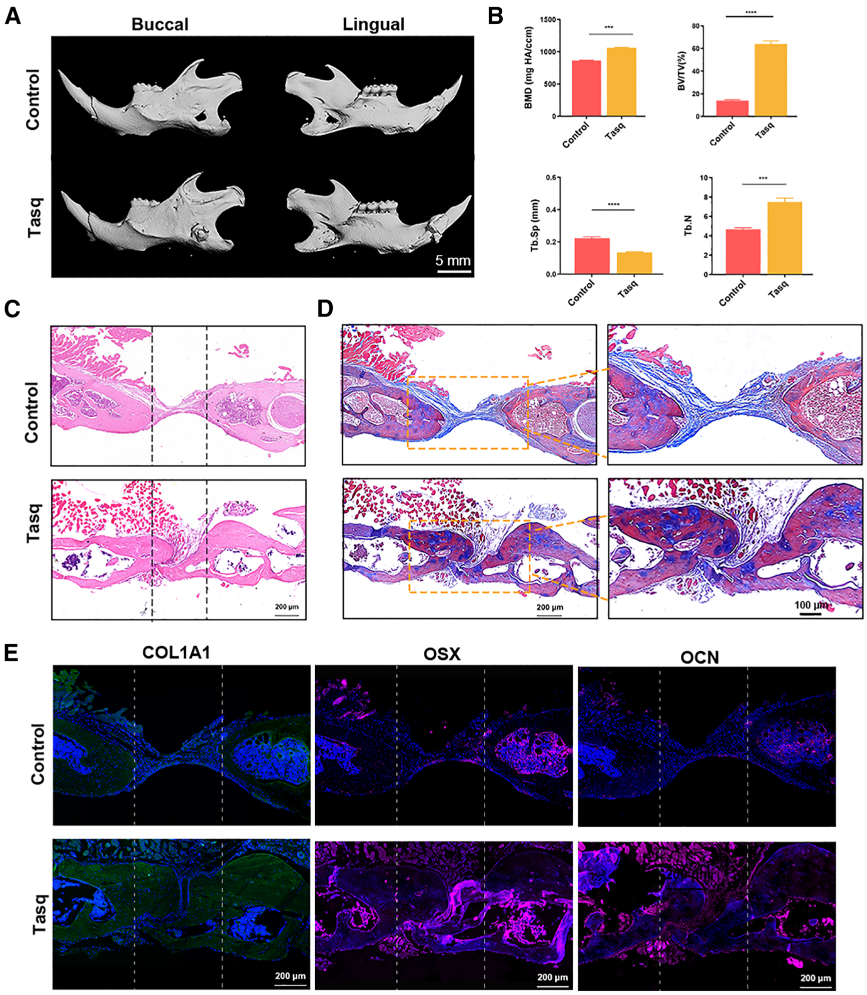

Figure 7. GelMA-F127 loaded with tasquinimod promotes mandibular bone defect repair

(A) Three-dimensional reconstruction of the mandibles 8 weeks after treatment with GelMA-F127 (control group) or GelMA-F127 loaded with tasquinimod (Tasq group; 1μM). Scale bars: 5 mm.

(B) Measurement of the ratio of bone volume/total volume (BV/TV), the bone mineral density (BMD), the trabecular number (Tb.N), and the trabecular spacing (Tb.Sp) in control versus Tasq groups. Data are mean ± SD (n = 5 mice per group from three independent experiments). Statistical significance was determined by an unpaired two-tailed t test. ***$p < 0.001$; ****$p < 0.0001$.

(C and D) Hematoxylin and eosin staining and Masson staining of the defect area in control versus Tasq groups. Scale bars: 200 μm (overview) and 100 μm (magnified view).

(E) Immunofluorescence staining of the osteogenic markers COL1A1, OSX, and OCN. Blue fluorescence, DAPI; green fluorescence, COL1A1; red fluorescence, OSX or OCN. Scale bars: 200 μm.

the Tasq group, with a marked reduction in Tb.Sp (Figure 7B). Histological and IF staining of COL1A1, OSX, and OCN (Figures 7C–7E) further confirmed enhanced new bone maturation and osteogenic marker expression. These results indicated that the GelMA-F127 hydrogel loaded with Tasq not only promotes hASC osteogenesis *in vitro* but also effectively enhances bone regeneration *in vivo*, demonstrating promising potential for bone tissue engineering applications.

DISCUSSION

Bone defect repair, particularly craniofacial reconstruction, remains a significant clinical challenge. The elucidation of stem cell osteogenic differentiation mechanisms and the development of targeted regulatory materials have now shifted to become a major research focus in the field of bone tissue engineering. A previous study showed that circ_0003204 regulates hASC osteogenic differentiation via the miR-370-3p-HDAC4 axis, identifying HDAC4 as a pivotal regulator in the osteogenic signaling network (Yu et al., 2022). However, the role of HDAC4 in regulating osteogenesis-related gene expression via histone acetylation and its impact on hASC osteogenic differentiation remain unclear.

In this study, we combined CUT&Tag with functional validation, which revealed that increased AcH3 peaks in the regulatory regions of key osteogenic regulators, including *AKT2*, *MAP2K2*, *RUNX2*, and *SMAD4*, were directly correlated with elevated mRNA expression of these genes. Further mechanistic investigation demonstrated that HDAC4 directly binds to the *SMAD4* promoter region. HDAC4 inhibition increased AcH3 peaks at the *SMAD4* promoter, thereby promoting both its protein expression and mRNA transcription. The consistency between promoter acetylation peaks and gene expression changes strongly supports our core hypothesis that HDAC4

inhibition relieves transcriptional repression by specifically increasing histone acetylation at osteogenic gene, thereby driving the osteogenic differentiation program. We note that future whole-transcriptome RNA sequencing (RNA-seq) would provide a more unbiased perspective on the transcriptional network modulated by HDAC4.

Our research demonstrated, for the first time, that HDAC4 suppresses hASC osteogenic differentiation by negatively regulating SMAD4 expression through modulation of histone H3 acetylation. HDAC4 knockdown significantly increased acetylation levels at the SMAD4 promoter region, concomitant with upregulation of its transcription and protein expression, suggesting that SMAD4 may serve as a direct epigenetic target of HDAC4 in osteogenic regulation. As a key transcription factor in the TGF-β pathway (Rasti et al., 2021), SMAD4 is essential for bone growth and development (Pakravan et al., 2022) and plays a crucial role in hASC osteogenic differentiation (Park et al., 2019). On the basis of our findings, we hypothesize that upregulating SMAD4 promotes hASC osteogenic differentiation, though further research is needed to clarify the mechanisms.

In recent years, accumulating evidence has highlighted the multidimensional regulatory role of the HDAC family in bone metabolism (Guttzeit and Backs, 2022). For instance, miR-29b enhances osteogenesis of BMSCs by suppressing HDAC4 (Liu et al., 2019), while miR-29a elevates RUNX2 and VEGF expressions through HDAC4 downregulation, synergistically promoting bone formation and vascularization (Pan et al., 2024). Conversely, TGF-β1 inhibits Nr4a1 expression by upregulating HDAC4, thereby inhibiting osteogenesis (Gao et al., 2024). Collectively, these studies demonstrate that HDAC4 serves as a conserved negative regulator in osteogenic differentiation. Our research further validates that HDAC4 knockdown enhances the osteogenic capacity of seed cells in both calvarial and mandibular defect models *in vivo*, laying the groundwork for its clinical translation.

Notably, although HDAC inhibitors (HDACi) are widely used in cancer therapy, their application in bone tissue engineering remains exploratory. We employed the HDAC4-specific inhibitor Tasq to evaluate, for the first time, its effects on hASC osteogenic potential and epigenetic modifications. The results demonstrated that 1 μM Tasq not only promotes histone acetylation but also significantly upregulates SMAD4 expression and increases the levels of osteogenic markers such as ALP and OSX, exhibiting effects highly consistent with HDAC4 knockdown. Compared with broad-spectrum HDACi such as vorinostat or romidepsin, Tasq demonstrated significantly higher specificity by targeting the Zn^{2+}-binding domain of HDAC4 (Fan et al., 2023; Uzdensky and Demyanenko, 2021). Recent studies have found that Tasq in 3D polycaprolactone and nano-hydroxyapatite inhibited angiogenesis by suppressing CAV-1 and deactivating the Wnt/β-catenin pathway (Wu et al., 2024). However, research on Tasq in osteogenesis is limited, and this study is the first to investigate its use in mandibular defect repair.

In the field of bone regeneration, preclinical studies have highlighted the multifaceted actions of broad-spectrum HDACi such as vorinostat and Trichostatin A (TSA). Under optimized dosing, vorinostat promotes osteogenesis through epigenetic reprogramming while concurrently inhibiting bone resorption by upregulating RUNX2 expression (Xu et al., 2013). Similarly, TSA modulates the osteoimmune microenvironment, combining anti-inflammatory and pro-regenerative effects (Kim et al., 2025). However, this broad-spectrum activity is a major drawback, leading to a narrow therapeutic window and increased risks of systemic toxicity and adverse effects. In contrast, the highly selective HDAC4 inhibitor Tasq offers a distinct advantage through its precise targeting. By specifically inhibiting HDAC4, it demonstrates the potential to preserve therapeutic efficacy while markedly reducing off-target effects, thereby providing promising long-term safety and tolerability. Tasq, thus, represents a novel strategic paradigm. Nevertheless, its efficacy in bone regeneration awaits systematic validation in relevant preclinical models of skeletal diseases.

To achieve targeted delivery of Tasq to local bone defects, we developed a GelMA-F127-Tasq composite system based on GelMA hydrogel. Porous GelMA with photocuring properties exhibits excellent injectability and biocompatibility *in vitro* and *in vivo*. Its porous structure facilitates biomolecule diffusion, making it ideal for drug delivery (Davoodi et al., 2022). However, as a hydrophilic carrier, GelMA encounters challenges in efficiently loading and releasing hydrophobic drugs (Malik et al., 2017). Polyether F127, a triblock co-polymer with polyethylene oxide segments and polypropylene oxide ends, improves drug delivery and hydrogel construction due to its mechanical properties including strength and toughness (Li et al., 2023; McAfee et al., 2021). The incorporation of F127 significantly enhanced the hydrophobic drug-loading capacity of GelMA while simultaneously reinforcing its mechanical strength. Material characterization confirmed that the GelMA-F127 hydrogel exhibits a continuously porous structure, high compressive modulus, and controllable degradation profile, enabling sustained Tasq release for localized modulation of osteogenesis.

Previous studies suggest that SMAD4 interacts with osteogenic transcription factors (e.g.,.RUNX2 and Sp7) to co-regulate osteogenic differentiation (Aspera-Werz et al., 2019). Our CUT&Tag and motif analysis revealed that the histone acetylation peaks regulated by HDAC4 are highly enriched in RUNX2- and SMAD4-binding motifs. Furthermore, HDAC4 inhibition (via knockdown or Tasq treatment) synchronously upregulates SMAD4 and

RUNX2 at both mRNA and protein levels. This co-expression pattern provides a prerequisite for synergistic regulation by these transcription factors. It is known that SMAD4 and RUNX2 exhibit crosstalk and synergistic effects in regulating osteogenic genes (Dai et al., 2026). We, therefore, speculate that during the osteogenic differentiation of hASCs, SMAD4 may co-occupy the promoter regions of downstream genes with RUNX2, thereby cooperatively activating transcription. Future research utilizing techniques such as ChIP and coIP to elucidate their protein-protein interaction and genomic co-localization will further clarify this regulatory network.

In conclusion, this study systematically reveals that HDAC4 inhibits the expression of osteogenic master regulators (e.g., SMAD4) by suppressing histone acetylation during hASC osteogenic differentiation. Both HDAC4 knockdown and its selective inhibitor Tasq significantly enhance histone acetylation, upregulate SMAD4 expression, and consequently promote osteogenic differentiation in hASCs. Validated via a localized delivery system incorporating GelMA-F127 composite hydrogel, this strategy substantially improved bone regeneration in calvarial and mandibular defects. These findings not only elucidate the epigenetic mechanism underlying HDAC4-mediated osteogenic regulation but also provide novel theoretical foundations and material-based approaches for targeted HDAC4 modulation in craniofacial bone regeneration.

RESOURCE AVAILABILITY

Lead contact

Requests for further information, resources, and reagents should be directed to and will be fulfilled by the lead contact, Jianwei Chen (chenjianwei2017@vip.163.com).

Materials availability

This study did not generate new unique reagents.

Data and code availability

Sequencing data generated in this study are deposited in GEO database, under the accession numbers GEO: GSE308788 (CUT&Tag sequencing).

ACKNOWLEDGMENTS

This study was supported by grants from the National Natural Science Foundation of China (82271017), fundamental research program funding of Ninth People's Hospital affiliated to Shanghai Jiao Tong University School of Medicine (JYZZ217), and Sichuan Provincial Natural Science Foundation (2025ZNSFSC0757).

AUTHOR CONTRIBUTIONS

L.Y. and K.X. contributed to conception, design, acquisition, and drafting and critical revision of the manuscript; Y.W. contributed to conception, analysis, and drafting and critical revision of the manuscript; Z.H. contributed to conception and analysis and drafted and critically revised the manuscript; J.L. and S.Z. contributed to conception, design, analysis, and critical revision of the manuscript; J.C. contributed to conception, design, acquisition, analysis, interpretation, and critical revision of the manuscript. All authors gave final approval and agree to be accountable for all aspects of the work.

DECLARATION OF INTERESTS

The authors declare that they have no competing interests.

STAR★METHODS

Detailed methods are provided in the online version of this paper and include the following:

- KEY RESOURCES TABLE
- EXPERIMENTAL MODEL AND STUDY PARTICIPANT DETAILS
  - Cell lines and culture conditions
  - Animals
- METHOD DETAILS
  - Osteogenic induction and assessment
  - siRNA transfecting
  - CUT&Tag sequencing
  - Differential peak and functional analysis
  - IGV visualization and motif analysis
  - RT-qPCR
  - CUT&Tag-qPCR
  - Chromatin immunoprecipitation
  - Immunofluorescence (IF) staining of cells and quantification
  - Western blot and quantification
  - Wound healing assay
  - Live/dead cell staining
  - CCK-8 assay
  - Apoptosis assay
  - Synthetic scaffold
  - SEM and EDS elemental mapping
  - High-performance liquid chromatography
  - Mechanical characterization
  - Swelling Assay and degradation test
  - *In vivo* biocompatibility tests
  - Establishment of a cranial defect model
  - Establishment of a mandibular bone defect model
  - Micro-CT analysis
  - Hematoxylin and eosin staining (H&E) staining
  - Masson's trichrome staining
  - If analysis of tissue sections
- QUANTIFICATION AND STATISTICAL ANALYSIS

SUPPLEMENTAL INFORMATION

Supplementary data related to this article can be found online at https://doi.org/10.1016/j.stemcr.2026.102955.

Received: January 6, 2026
Revised: May 12, 2026
Accepted: May 12, 2026
Published: June 11, 2026

REFERENCES

Aspera-Werz, R.H., Chen, T., Ehnert, S., Zhu, S., Fröhlich, T., and Nussler, A.K. (2019). Cigarette Smoke Induces the Risk of Metabolic Bone Diseases: Transforming Growth Factor Beta Signaling Impairment via Dysfunctional Primary Cilia Affects Migration, Proliferation, and Differentiation of Human Mesenchymal Stem Cells. Int. J. Mol. Sci. *20*, 2915. https://doi.org/10.3390/ijms20122915.

Blixt, N.C., Faulkner, B.K., Astleford, K., Lelich, R., Schering, J., Spencer, E., Gopalakrishnan, R., Jensen, E.D., and Mansky, K.C. (2017). Class II and IV HDACs function as inhibitors of osteoclast differentiation. PLoS One *12*, e0185441. https://doi.org/10.1371/journal.pone.0185441.

Brennen, W.N., Nguyen, H., Dalrymple, S.L., Reppert-Gerber, S., Kim, J., Isaacs, J.T., and Hammers, H. (2016). Assessing angiogenic responses induced by primary human prostate stromal cells in a three-dimensional fibrin matrix assay. Oncotarget *7*, 71298–71308. https://doi.org/10.18632/oncotarget.11347.

Chen, C., Zong, S., Wang, Z., Yang, R., Guo, Y., Wang, Y., Chen, X., Li, Y., and Wang, S. (2023). FTY720 Attenuates LPS-Induced Inflammatory Bone Loss by Inhibiting Osteoclastogenesis via the NF-κB and HDAC4/ATF Pathways. J. Immunol. Res. *2023*, 1–14. https://doi.org/10.1155/2023/8571649.

Dai, X., Wang, Y., Huang, X., Meng, Z., and Zheng, P. (2026). Current advances of bone homeostasis imbalance in the cause of hereditary metabolic bone diseases. EFORT Open Rev. *11*, 72–84. https://doi.org/10.1530/eor-2025-0147.

Davoodi, E., Montazerian, H., Zhianmanesh, M., Abbasgholizadeh, R., Haghniaz, R., Baidya, A., Pourmohammadali, H., Annabi, N., Weiss, P.S., Toyserkani, E., and Khademhosseini, A. (2022). Template-Enabled Biofabrication of Thick 3D Tissues with Patterned Perfusable Macrochannels. Adv. Healthcare Mater. *11*, e2102123. https://doi.org/10.1002/adhm.202102123.

Fan, R., Satilmis, H., Vandewalle, N., Verheye, E., Vlummens, P., Maes, A., Muylaert, C., De Bruyne, E., Menu, E., Evans, H., et al. (2023). Tasquinimod suppresses tumor cell growth and bone resorption by targeting immunosuppressive myeloid cells and inhibiting c-MYC expression in multiple myeloma. J. Immunother. Cancer *11*, e005319. https://doi.org/10.1136/jitc-2022-005319.

Gao, Y., Zou, Y., Sokolowskei, D., Xing, X., Tower, R.J., Lai, Z., Shi, J., Zhu, L., Zheng, Q., James, A.W., et al. (2024). Nr4a1 enhances Wnt4 transcription to promote mesenchymal stem cell osteogenesis and alleviates inflammation-inhibited bone regeneration. Mol. Ther. *32*, 1479–1496. https://doi.org/10.1016/j.ymthe.2024.02.034.

Gomathi, K., Akshaya, N., Srinaath, N., Rohini, M., and Selvamurugan, N. (2021). Histone acetyl transferases and their epigenetic impact on bone remodeling. Int. J. Biol. Macromol. *170*, 326–335. https://doi.org/10.1016/j.ijbiomac.2020.12.173.

Guan, Y., Chen, Q., Yang, X., Haines, P., Pei, M., Terek, R., Wei, X., Zhao, T., and Wei, L. (2012). Subcellular relocation of histone deacetylase 4 regulates growth plate chondrocyte differentiation through Ca2+/calmodulin-dependent kinase IV. Am J Physiol Cell Physiol *303*, C33–C40. https://doi.org/10.1152/ajpcell.00348.2011.

Guttzeit, S., and Backs, J. (2022). Post-translational modifications talk and crosstalk to class IIa histone deacetylases. J. Mol. Cell. Cardiol. *162*, 53–61. https://doi.org/10.1016/j.yjmcc.2021.08.007.

Isaacs, J.T., Antony, L., Dalrymple, S.L., Brennen, W.N., Gerber, S., Hammers, H., Wissing, M., Kachhap, S., Luo, J., Xing, L., et al. (2013). Tasquinimod Is an Allosteric Modulator of HDAC4 survival signaling within the compromised cancer microenvironment. Cancer Res. *73*, 1386–1399. https://doi.org/10.1158/0008-5472.Can-12-2730.

Kim, H., Kang, L., and Park, S.Y. (2025). HDAC inhibitors restore osteoimmune balance and bone regeneration via selective MAPK modulation in inflammatory bone disease. Stem Cell Res. Ther. *16*, 604. https://doi.org/10.1186/s13287-025-04719-2.

Li, P., and Guo, X. (2018). A review: therapeutic potential of adipose-derived stem cells in cutaneous wound healing and regeneration. Stem Cell Res. Ther. *9*, 302. https://doi.org/10.1186/s13287-018-1044-5.

Li, S., Yang, C., Li, J., Zhang, C., Zhu, L., Song, Y., Guo, Y., Wang, R., Gan, D., Shi, J., et al. (2023). Progress in Pluronic F127 Derivatives for Application in Wound Healing and Repair. Int J Nanomedicine *18*, 4485–4505. https://doi.org/10.2147/ijn.S418534.

Liu, Q., Lin, Z., Liu, Y., Du, J., Lin, H., and Wang, J. (2019). Delivery of miRNA-29b Using R9-LK15, a Novel Cell-Penetrating Peptide, Promotes Osteogenic Differentiation of Bone Mesenchymal Stem Cells. Biomed Res. Int. *2019*, 1–12. https://doi.org/10.1155/2019/3032158.

Magnusson, L.U., Hagberg Thulin, M., Plas, P., Olsson, A., Damber, J.E., and Welén, K. (2016). Tasquinimod inhibits prostate cancer growth in bone through alterations in the bone microenvironment. Prostate *76*, 383–393. https://doi.org/10.1002/pros.23133.

Malik, N.S., Ahmad, M., and Minhas, M.U. (2017). Cross-linked β-cyclodextrin and carboxymethyl cellulose hydrogels for controlled drug delivery of acyclovir. PLoS One *12*, e0172727. https://doi.org/10.1371/journal.pone.0172727.

McAfee, T., Ferron, T., Cordova, I.A., Pickett, P.D., McCormick, C.L., Wang, C., and Collins, B.A. (2021). Label-free characterization of organic nanocarriers reveals persistent single molecule cores for hydrocarbon sequestration. Nat. Commun. *12*, 3123. https://doi.org/10.1038/s41467-021-23382-8.

Pakravan, K., Razmara, E., Mahmud Hussen, B., Sattarikia, F., Sadeghizadeh, M., and Babashah, S. (2022). SMAD4 contributes to chondrocyte and osteocyte development. J. Cell Mol. Med. *26*, 1–15. https://doi.org/10.1111/jcmm.17080.

Pan, S., Yin, Z., Shi, C., Xiu, H., Wu, G., Heng, Y., Zhu, Z., Zhang, J., Gui, J., Yu, Z., and Liang, B. (2024). Multifunctional Injectable Hydrogel Microparticles Loaded with miR-29a Abundant BMSCs Derived Exosomes Enhanced Bone Regeneration by Regulating Osteogenesis and Angiogenesis. Small *20*, 2306721. https://doi.org/10.1002/smll.202306721.

Park, J.S., Kim, M., Song, No-J., Kim, Jun-H., Seo, D., Lee, Ji-H., Jung, S.M., Lee, J.Y., Lee, J., Lee, Y.S., et al. (2019). A Reciprocal Role of the Smad4-Taz Axis in Osteogenesis and Adipogenesis of Mesenchymal Stem Cells. Stem Cell. *37*, 368–381. https://doi.org/10.1002/stem.2949.

Rasti, A., Madjd, Z., Zanjani, L.eili S.aeednejad, Babashah, S.adegh, Abolhasani, M.aryam, Asgari, M.ojgan, and Mitra, M.ehrazma (2021). SMAD4 Expression in Renal Cell Carcinomas Correlates With a Stem-Cell Phenotype and Poor Clinical Outcomes. Front. Oncol. *11*, 581172. https://doi.org/10.3389/fonc.2021.581172.

Uzdensky, A., and Demyanenko, S. (2021). Histone acetylation and deacetylation in ischemic stroke. Neural Regen. Res. *16*, 1529–1530. https://doi.org/10.4103/1673-5374.303024.

Wang, Z., Qin, G., and Zhao, T.C. (2014). HDAC4: mechanism of regulation and biological functions. Epigenomics *6*, 139–150. https://doi.org/10.2217/epi.13.73.

Wu, D., Zheng, K., Yin, W., Hu, B., Yu, M., Yu, Q., Wei, X., Deng, J., and Zhang, C. (2024). Enhanced osteochondral regeneration with a 3D-Printed biomimetic scaffold featuring a calcified interfacial layer. Bioact. Mater. *36*, 317–329. https://doi.org/10.1016/j.bioactmat.2024.03.004.

Wu, T., Tang, H., Yang, J., Yao, Z., Bai, L., Xie, Y., Li, Q., and Xiao, J. (2022). METTL3-m(6) A methylase regulates the osteogenic potential of bone marrow mesenchymal stem cells in osteoporotic rats via the Wnt signalling pathway. Cell Prolif. *55*, e13234. https://doi.org/10.1111/cpr.13234.

Xu, S., De Veirman, K., Evans, H., Santini, G.C., Vande Broek, I., Leleu, X., De Becker, A., Van Camp, B., Croucher, P., Vanderkerken, K., and Van Riet, I. (2013). Effect of the HDAC inhibitor vorinostat on the osteogenic differentiation of mesenchymal stem cells in vitro and bone formation in vivo. Acta Pharmacol. Sin. *34*, 699–709. https://doi.org/10.1038/aps.2012.182.

Xu, Y., Yan, S., Chen, C., Lu, B., and Zhao, R. (2022). Constructing Injectable Bone-Forming Units by Loading a Subtype of Osteoprogenitors on Decellularized Bone Matrix Powders for Bone Regeneration. Front. Cell Dev. Biol. *10*, 910819. https://doi.org/10.3389/fcell.2022.910819.

Yu, L., Gao, T., Li, W., Yang, J., Liu, Y., Zhao, Y., He, P., Li, X., Guo, W., Fan, Z., and Dai, H. (2023). Carboxymethyl chitosan-alginate enhances bone repair effects of magnesium phosphate bone cement by activating the FAK-Wnt pathway. Bioact. Mater. *20*, 598–609. https://doi.org/10.1016/j.bioactmat.2022.06.017.

Yu, L., Xia, K., Zhou, J., Hu, Z., Yin, X., Zhou, C., Zou, S., and Liu, J. (2022). circ_0003204 regulates the osteogenic differentiation of human adipose-derived stem cells via miR-370-3p/HDAC4 axis. Int. J. Oral Sci. *14*, 30. https://doi.org/10.1038/s41368-022-00184-2.

STAR★METHODS

KEY RESOURCES TABLE

REAGENT or RESOURCE	SOURCE	IDENTIFIER
**Antibodies**		
Anti-AcH3	Millipore	Cat# 06-599; RRID:AB_2115283
Anti-HDAC4	Proteintech (China)	Cat# 66838-1-Ig, RRID:AB_2882181
Anti-HDAC5	Affinity (China)	Cat# AF5348, RRID:AB_2837833
Anti-HDAC7	Affinity (China)	Cat# AF0180, RRID:AB_2833373
Anti-COL1A1	Cell Signaling Technology (USA)	Cat# 72026, RRID:AB_2904565
Anti-RUNX2	ServiceBio (China)	Cat# GB13264, RRID:AB_3676299
Anti -SMAD4	Huabio (China)	Cat# ET1604-12, RRID:AB_3073456
Anti -ALP	Huabio (China)	Cat# ET1601-21, RRID:AB_3069604
Anti-OSX	Abcam (Cambridge, UK)	Cat# ab209484, RRID:AB_2892207
Anti-OCN	ServiceBio (China)	Cat# GB11233, RRID:AB_3676307
GAPDH	Huabio (China)	Cat# R1208-3, RRID:AB_3712825
ACTIN	ServiceBio (China)	Cat# GB15001, RRID:AB_3083704
Goat anti-Rabbit IgG-Alexa Flour 488® antibody	Thermo Fisher Scientific (USA)	Cat# A-11008, RRID:AB_2534119
Goat anti-Mouse IgG-Alexa Flour 594® antibody	Thermo Fisher Scientifc (USA)	Cat# A-11005, RRID:AB_2534093
**Biological samples**		
hASCs	Cyagen (China)	Cat# HUXMD-01001
**Chemicals, peptides, and recombinant proteins**		
Tasquinimod	Solarbio (China)	Cat# IT1560
Lipofectamine 3000 Reagent	Thermo Fisher Scientific (USA)	Cat# L3000015
Opti-MEM Reduced Serum Medium	Thermo Fisher Scientific (USA)	Cat# 31985062
GelMA	Engineering For Life (China)	Cat# EFL-GM-90
Polyether F127	Engineering For Life (China)	Cat# EFL-F127
OriCell™ Human Adipose-derived MSC Osteogenic Differentiation Medium	Cyagen (China)	Cat# HUXMD-90021
IP buffer	Servicebio (China)	Cat# G2038-100ML
Proteinase K	Servicebio (China)	Cat# G1234-1ML
Protein A/G Antibody Purification Magnetic Beads	Servicebio (China)	Cat# G3663-5ML
DAPI	SolarBio (China)	Cat# S2110; RRID: N/A
**Critical commercial assays**		
RNAiso Plus kit	Takara (Japan)	Cat# 9108

(*Continued on next page*)

Continued

REAGENT or RESOURCE	SOURCE	IDENTIFIER
RevertAid First Strand cDNA Synthesis Kit	Thermo Fisher Scientific (USA)	Cat# K16225
TB Green Premix Ex Taq II	Takara (Japan)	Cat# RR820A
CCK-8 Kit	Dojindo (China)	Cat# CK04-01
Live/Dead Cell Staining Kit	Keygenbio (China)	Cat# KGAF001
Annexin V-FITC Apoptosis Detection Kit	Dojindo (China)	Cat# AD10
CUT&Tag Assay Kit	Vazyme (China)	Cat# TD903
Masson's Trichrome Stain Kit	Solarbio (China)	Cat# G1340
**Deposited data**		
CUT&Tag sequencing data	This paper	GEO: GSE308788
**Experimental models: Organisms/strains**		
Rat: SD rat (Sprague-Dawley)	Chengdu Dashuo Co. (China)	N/A
Mouse: BALB/c nude mouse	Jiangsu Jicui Yaokang Biotechnology (China)	N/A
Mouse: C57BL/6 mouse	Chengdu Dashuo Co. (China)	N/A
**Oligonucleotides**		
si-HDAC4 and si-NC sequences	This paper	See Table S1
Primers for RT-qPCR	This paper	See Table S2
Primers for CUT&Tag-qPCR	This paper	See Table S3
Primers for Human-specific Alu	This paper	See Table S4
**Software and algorithms**		
fastp (v0.20.0)	N/A	https://github.com/OpenGene/fastp
BWA (v0.7.12)	N/A	http://bio-bwa.sourceforge.net/
MACS2 (v2.1.0)	N/A	https://github.com/macs3-project/MACS
ChIPseeker (Bioconductor package)	N/A	https://bioconductor.org/packages/release/bioc/html/ChIPseeker.html
HOMER (v4.9.1)	N/A	http://homer.ucsd.edu/homer/
KOBAS (KEGG Orthology Based Annotation System)	N/A	http://kobas.cbi.pku.edu.cn/
ImageJ (v6.0)	National Institutes of Health	https://imagej.nih.gov/ij/
GraphPad Prism	GraphPad Software	https://www.graphpad.com/
IGV (Integrative Genomics Viewer)	Broad Institute	https://igv.org/
**Other**		
Ethical Approval	West China School of Stomatology, Sichuan University	WCHSIRB-D-2022-596

EXPERIMENTAL MODEL AND STUDY PARTICIPANT DETAILS

Cell lines and culture conditions

Human adipose-derived mesenchymal stem cells (hASCs; OriCell, HUXMD-01001, Cyagen, China) were cultured in complete medium. Cells between passages 3 and 7 were used for all subsequent experiments. For osteogenic differentiation,

the culture medium was replaced with OriCell human adipose-derived mesenchymal stem cell osteogenic differentiation medium (Cyagen, China), which was changed every three days. Osteogenic differentiation was assessed after 7 and 14 days of induction.

Animals

All animal procedures were approved by the Ethics Committee of West China School of Stomatology, Sichuan University (WCHSIRB-D-2022-596). Eight-week-old SPF-grade male Sprague-Dawley (SD) rats were purchased from Chengdu Dashuo Company (China) for *in vivo* biocompatibility tests. Six-week-old female BALB/c nude mice and six-week-old male C57BL/6 mice were purchased from Jiangsu Jicui Yaokang Biotechnology (Nanjing, China) for cranial and mandibular defect models. Animals were housed under standard conditions with free access to food and water.

METHOD DETAILS

Osteogenic induction and assessment

Osteogenic differentiation of hASCs was induced as described above. Alkaline phosphatase (ALP) staining was performed 7 days after osteogenic induction. First, rinse the cells with PBS three times. Fix the cells with citric acid-acetone-formaldehyde fixative for 30 s. After fixation, wash with distilled water three times. Add 0.5mL of prepared staining solution to each well. Allow to react fully in the dark for 15 min, remove the staining solution, and rinse with distilled water three times. Finally, observe the staining effect under a light microscope. Alizarin Red S (ARS) staining was performed 14 days after osteogenic induction. As described in the instructions, cells were washed with PBS three times and fixed with 4% paraformaldehyde for 30 min. After fixation, 0.5mL of Alizarin Red S staining solution was added to each well and allowed to react for 3-5 min. After staining, the stained wells were observed under a microscope for the staining effect.

siRNA transfecting

First, $2 \times 10^{\hat{}6}$ cells were seeded in a 24-well plate and divided into the knockdown (si-HDAC4) and the control (si-NC) groups. Cells were transfected when they reached 80% confluence. For transfection, 100 μL of opti-MEM and 3 μL of Lip3000 were combined in an EP tube as Solution A. In a new EP tube, 100 μL of opti-MEM, 2 μg of RNA, and 4 μL of P3000 were mixed to form Solution B. Solutions A and B were combined and incubated at room temperature for 5 min. Then, 50 μL of the transfection mixture was added to each sample. After 24 h, the medium was changed, and transfection efficiency was assessed after 48 h. The sequences are shown in Table S1.

CUT&Tag sequencing

CUT&Tag assays was performed using an Illumina kit and anti-AcH3 antibody (Cat# 06-599, Millipore). Libraries were purified with AMPure beads, quality-checked on an Agilent Bioanalyzer 2100, and sequenced by Novogene (Beijing) on an Illumina platform (TruSeq PE Cluster Kit), generating 150 bp raw reads. Raw reads were processed with fastp (v0.20.0) to obtain clean reads (Q20/Q30/GC metrics calculated). Clean reads were aligned to a reference genome (downloaded from public databases) using BWA (v0.7.12) with MAPQ $\geq$13 filtering. Peak calling was performed with MACS2 (v2.1.0) under parameters: q 0.05, f AUTO, call-summits, nomodel, shift 100, extsize 200, keep-dup all. Significant peaks ($p < 0.05$) were retained for downstream analysis.

Differential peak and functional analysis

Peaks from different groups were merged, and the average RPM for each group was calculated. Differential Peaks were filtered with an RPM $\geq$2. The related genes of the differential Peaks were identified using the ChIPseeker technology. The data were exported and visually compared using cluster analysis and Venn diagrams. GO functional enrichment analysis was performed on these genes ($p < 0.05$). KEGG pathway analysis was conducted using KOBAS software to predict enrichment pathways.

IGV visualization and motif analysis

IGV is a powerful genome visualization tool that can be used to analyze and visualize various biological data, such as chromosomes, genes, exons, and transcripts. Histone acetylation data and reference genome annotation files were loaded into the software. In the chromosome pane of IGV, the chromosome of interest was selected for analysis, and the display range was set to view the genes and annotation information on the chromosome. A Motif is a typical sequence segment that represents the sequence conservation at the peak summit, indicating interactions with DNA base sequences and playing

a role in the regulation of gene expression. Therefore, we conducted a Motif analysis of HDAC4 histone acetylation modification sites to understand how histone acetylation modifications influence protein function. First, sequences 3 kb upstream and downstream of the acetylation sites were extracted from the genomic or transcript sequences. The bioinformatics tool HOMER (version 4.9.1) was used to identify conserved sequences associated with histone acetylation and generate a Motif graphic to display the frequency of corresponding bases at that position.

RT-qPCR

RT-qPCR was performed using the RNAiso Plus kit (Takara, Japan) to extract total RNA, and the purity and concentration of RNA are detected using NanoDrop spectrophotometer (NanoDrop Technologies, USA). Subsequently, the total RNA is reverse transcribed into cDNA using RevertAid First Strand cDNA (SynthesThermo Fisher, USA). The ABI 7500 Fast Real-Time PCR System (Applied Biosystems) is used for qPCR detection. Relative quantification is normalized and calculated using GAPDH in the $2^{-\Delta\Delta Ct}$ formula. All primers are listed in Table S2.

CUT&Tag-qPCR

The DNA sequences near the target protein were obtained using the CUT&Tag Assay Kit under the guidance of anti-histone H3 acetylation (AcH3). The qPCR procedure is as described above. Primers are listed in Table S3.

Chromatin immunoprecipitation

Chromatin immunoprecipitation (ChIP) was performed to validate the direct binding of HDAC4 to the SMAD4 promoter region. Cells were cross-linked with 1% formaldehyde for 10 min at 37°C, and the reaction was quenched with 125 mM glycine. Following lysis in IP buffer containing protease inhibitors, chromatin was sheared by sonication to obtain fragments ranging from 200 to 1000 bp. A portion of the lysate was reserved as the Input control. The remaining chromatin was precleared with Protein A/G magnetic beads and salmon sperm DNA for 1 h at 4°C. Immunoprecipitation was carried out overnight at 4°C using 1 μg of specific anti-HDAC4 antibody (Proteintech, wuhan, China) or an equivalent amount of control IgG. Antibody–chromatin complexes were captured with Protein A/G magnetic beads for 2 h, followed by sequential washes with low-salt, high-salt, LiCl, and TE buffers. Complexes were eluted in freshly prepared elution buffer (1% SDS, 0.1 M $NaHCO_3$. To reverse cross-linking, NaCl (final 0.2 M) and proteinase K were added, and samples were incubated overnight at 55°C. DNA was purified using a magnetic bead–based cleanup kit and eluted in nuclease-free water. Enrichment of the SMAD4 promoter region was quantified by real-time PCR with the primers 5′-GACTGTCCTGTGCGTCTC-3′ (forward) and 5′-GAAGGTGGCTAGGTTGTAG-3′ (reverse). Enrichment was calculated relative to the Input control and normalized to the IgG control, confirming direct HDAC4 binding to the SMAD4 promoter.

Immunofluorescence (IF) staining of cells and quantification

Cells were fixed with 4% paraformaldehyde (30 min), permeabilized with 0.1% Triton X-100 (30 min), and blocked with 5% BSA (30 min). They were then incubated overnight at 4°C with anti-AcH3 (1:500; Millipore) and anti-COL1A1 (1:200; Cell Signaling Technology, America), followed by fluorescent secondary antibodies (Solarbio, China) for 2 h in the dark. Nuclei were stained with DAPI (Solarbio, China) for 10 min, and samples were imaged using a confocal microscope. Fluorescence intensity was quantified with ImageJ (version 6.0, National Institutes of Health).

Western blot and quantification

Total protein was extracted using RIPA (with 2 μL PMSF). The SDS-PAGE was prepared according to the manufacturer's instructions and used to separated proteins. The proteins were then transferred onto a PVDF membrane and incubated overnight in antibody dilution buffer: anti-SMDA4 (1:1000, Huabio, China), anti-ALP (1:500, Huabio, China), anti-OSX (1:1000, Abcam,UK), and anti-GAPDH (1:1000, Huabio, China). Subsequently, the membrane was incubated with the secondary antibody (1:5,000, Huabio, China) for 1 h, followed by light-protected incubation in the developing solution for 2-3 min before exposure. Finally, the band densities were analyzed using ImageJ.

Wound healing assay

Cells were seeded into a 6-well plate, with 2×10^5 cells per well. After incubation for approximately 24 h in an incubator (37°C, 5% CO_2) and reaching 100% confluence, a vertical scratch was made across the cell surface using a 200 μL pipette tip. The samples were divided into five groups according to the Tasquinimod (Tasq) concentration gradient (0, 1 μM, 2.5 μM, 5 μM, 10 μM). Tasq at the corresponding concentrations was added to serum-free medium, mixed thoroughly,

and then added to the wells. The plate was returned to the incubator, and microscope images were taken at different time points (0 h, 12 h, 24 h, 48 h).

Live/dead cell staining

Cells cultured in 24-well plates were treated with Tasq (0, 1, 2.5, 5, 10 μM) for 3 or 7 days. After treatment, cells were collected and stained using a Live/Dead Cell Staining Kit (Keygenbio, China). A live/dead staining working solution was prepared following manufacturer instructions. Cells were washed with PBS, stained with 200 μL working solution at 37°C in the dark for 30 min, washed again, and imaged using a confocal microscope (Olympus FV3000, Japan).

CCK-8 assay

Cells were seeded into a 96-well plate. According to the above method, the samples were divided into 5 groups (0, 1 μM, 2.5 μM, 5 μM, 10 μM). After culturing the cells for 1, 3, 5, and 7 days, cell proliferation was assessed using a CCK-8 kit (Dojindo, China). As the manufacturer's instructions, 20 μL of CCK-8 reagent was added to each well at the designated time points. The plate was gently shaken to mix, and then incubated at 37°C for 4 h. After incubation, the absorbance was measured at 405 nm.

Apoptosis assay

Cells were seeded into a 6-well plate. The samples were divided into 5 groups (0, 1 μM, 2.5 μM, 5 μM, 10 μM) according to the above method, and treated with the corresponding concentrations of Tasq. After 72 h of incubation, cells were collected using trypsin without EDTA and assessed for apoptosis using an apoptosis detection kit (Dojindo, China). Specifically, an apoptosis inducer with a final concentration of 1 g/mL was added to each sample and incubated at 37°C for 3.5 h. After centrifugation at 1,000 rpm for 3 min, pre-prepared 1×Annexin V Binding Solution was added. Then, 100 μL of cell suspension was mixed with 5 μL Annexin V-FITC conjugate and 5 μL PI Solution, and incubated in the dark for 15 min. Afterward, 400 μL of 1×Annexin V Binding Solution was added. Finally, the effect of different concentrations of Tasq on cell apoptosis was evaluated using a flow cytometer (Thermo Fisher Scientific).

Synthetic scaffold

GM-90 and F127 were purchased from EFL Company (Hangzhou, China). 10% (w/v) GelMA was synthesized according to the instructions. First, 10 mL of PBS was added to the LAP photoinitiator to prepare a 0.25% (w/v) photoinitiator solution. The required amount of GelMA powder for a 10% (w/v) solution was precisely weighed using analytical instruments. The mixture was then heated in a 65°C water bath for 30 min with slight agitation to ensure complete dissolution. Following this, GelMA-F127 was prepared by reheating 1 mL of the 10% (w/v) GelMA solution and adding 12 mg of F127 powder. After thorough mixing, GelMA-F127 was successfully synthesized, achieving a final F127 concentration of 1.2% (w/v). Finally, Tasq was added to the GelMA-F127 solution at a ratio of 1:1000, thoroughly mixed, and used for subsequent experiments.

SEM and EDS elemental mapping

SEM is a high-resolution microscopy technique that utilizes a focused electron beam to scan the surface of the sample, producing detailed morphological images. EDS, on the other hand, performs elemental analysis by detecting the characteristic X-rays emitted from the sample under electron beam excitation and generates elemental mapping, illustrating the spatial distribution of elements within the sample. The GelMA-F127-Tasq samples were synthesized and photocrosslinked as described, then frozen at −20°C and subsequently freeze-dried. Finally, the surface microstructure of the materials in each group was observed using SEM (Zeiss, Germany), and their elemental composition was analyzed by EDS (Oxford, UK).

High-performance liquid chromatography

The release profile of Tasq from GelMA-F127 hydrogel was evaluated by high-performance liquid chromatography (HPLC). Each hydrogel was incubated in 5 mL of PBS (pH 7.4) at 37°C under gentle agitation. At predetermined time points (0, 1, 3, 6, 12, 24, 48, and 72 h), the entire release medium was collected and filtered through a 0.22 μm membrane. Analysis was performed on a Waters 2695 HPLC system equipped with a C18 column (150 × 4.6 mm, 5 μm) maintained at 30°C. An isocratic mobile phase consisting of acetonitrile and 0.1% aqueous formic acid (45:55, v/v; ratio adjustable as needed) was used at a flow rate of 1.0 mL/min. Detection was carried out at 302 nm with an injection volume of 20 μL. Quantification was achieved using an external standard calibration curve. The concentration of Tasq in the release

medium at each time point was calculated based on the corresponding peak area, and the cumulative drug release was subsequently determined to characterize the release kinetics.

Mechanical characterization

GelMA and GelMA-F127 were synthesized as described. A 200 μL aliquot of each sample was placed into curing rings (EFL-SCR-3D-24, EFL Technology, China) and subjected to photocrosslinking. The cylindrical samples were immersed in PBS for 24 h to achieve swelling equilibrium. Compression modulus testing was then performed using an electronic universal testing machine (SANS, China) at a loading rate of 1 mm/min. The resulting data were analyzed, and stress-strain curves were generated.

Swelling Assay and degradation test

Hydrogel samples were prepared using curing rings and immersed in 2 mL of sterile PBS at 37°C. Samples were collected at 0, 1, 2, 4, 6, 12, 18, and 24 h, and the swollen mass (Ws) was measured. The samples were then pre-frozen at −20°C, freeze-dried, and their dry mass (Wf) was recorded. The swelling ratio was calculated as follows: Swelling ratio (100%) = (Ws - Wf)/Ws. For *in vitro* degradation tests, each group of samples was immersed in 2 mL of sterile PBS at 37°C for 24 h to reach swelling equilibrium. The samples were then transferred to sterile PBS containing Col II (2 U/mL) for further immersion. At 0, 1, 2, 4, and 6 h, the samples were freeze-dried, and their dry weight (Wd) was recorded. The dry weight at 0 h was denoted as Wd0, while the dry weights at subsequent time points were recorded as Wdn. The degradation rate was calculated as follows: Degradation rate (100%) = (Wd0 - Wdn)/Wdn.

In vivo biocompatibility tests

All animal procedures were approved by the Ethics Committee of West China School of Stomatology, Sichuan University (WCHSIRB-D-2022-596). GelMA-F127 was synthesized and loaded with Tasq according to the aforementioned method, followed by curing in curing rings. Eight-week-old SPF-grade male SD rats (Chengdu Dashuo Company, China) were randomly divided into two groups: the sham group (no material implanted) and the Tasq group (GelMA-F127 loaded with Tasq implanted). After sodium pentobarbital anesthesia, the left dorsal skin was incised, material implanted, and wounds sutured. At 2 weeks, blood was collected via tail clipping for hematological analysis (RBC, WBC, platelets). At 8 weeks, rats were euthanized by cervical dislocation; major organs (heart, liver, spleen, lungs, kidneys) were harvested, fixed in 4% paraformaldehyde (48 h), and processed for H&E staining after dehydration, embedding, and sectioning.

Establishment of a cranial defect model

According to the instructions, 10% (w/v) GelMA (Engineering For Life company, China) is prepared for cell loading. The cells transfect with si-HDAC4 and si-NC, and then seed them into GelMA to form microspheres with 4 mm diameter. Six-week-old female nude mice (BALB/c) were purchased from Jiangsu Jicui Yaokang Biotechnology (Nanjing, China). The experiment was divided into two groups: the si-HDAC4 group and the si-NC group. The mice were anesthetized with isoflurane inhalation. A gingival circular punch (inner diameter 3 mm, outer diameter 4 mm) was used to create a critical-sized bone defect with a diameter of 4 mm on the left side of the cranial suture. The prepared cell-scaffold microspheres were implanted into the defect site. Eight weeks after the surgery, the nude mice were sacrificed. The skull samples were harvested and fixed in 4% paraformaldehyde solution for further study.

Establishment of a mandibular bone defect model

Six-week-old female nude mice (BALB/c) were randomly assigned to two groups: the si-HDAC4 group and the si-NC group. Similarly, six-week-old male C57BL/6 mice (Chengdu Dashuo Company, China) were randomly divided into two groups: the Tasq group received GelMA-F127-Tasq, and the control group received GelMA-F127 alone. Surgical procedures (Figure 3A) included 1 cm left cheek incision with soft tissue dissection, creation of 2.3 mm mandibular defect using dental drill, hydrogel injection with light curing, and layered closure using 5-0 sutures. Mandibles were harvested 8 weeks post-surgery, PBS-rinsed, and fixed in 4% paraformaldehyde (48 h) for subsequent analysis.

Micro-CT analysis

The prepared skull samples were fixed in the scanning tube and placed into the micro-CT scanning chamber. A three-dimensional scan was performed using a micro-CT (Scanco, Switzerland) with a voxel size of 10 μm and a voltage of 70 kVp/200 μA. Data was collected, followed by three-dimensional reconstruction and image export. Bone data analysis

was conducted on the bone defect area, including the calculation of bone volume/total volume (BV/TV), bone mineral density (BMD), the trabecular thickness (Tb.Th), the trabecular spacing (Tb.Sp) and trabeculae number (Tb.N).

Hematoxylin and eosin staining (H&E) staining
The samples were immersed in hematoxylin staining solution for 3 min, and the nuclear staining was observed under a microscope. Differentiation was carried out in 1% hydrochloric acid alcohol for 2 s, followed by bluing in ammonia water for 1 min. Finally, the samples were immersed in eosin staining solution for 1 min. After rinsing with running water, the cytoplasmic staining was observed under the microscope After staining, the sections were dehydrated in a graded series of ethanol (70%, 85%, 90%, 100%, 100%). The sections were then cleared in xylene I and xylene II and mounted with neutral resin.

Masson's trichrome staining
The samples were stained using Masson's Trichrome Stain Kit (Solarbio, China). According to the manufacturer's instructions, samples incubated in solution A (room temperature, overnight), treated sequentially with solution B/C mix (3 min), 1% hydrochloric acid alcohol (2 s), solution D (6 min), solution E (1 min), solution F (10 s), then differentiated in 1% glacial acetic acid baths (8 s each) for three times, followed by dehydration, clearing, and microscopic observation.

If analysis of tissue sections
Tissue sections underwent antigen retrieval in sodium citrate buffer (Beyotime) at 98°C for 25 min, blocked with goat serum (30 min). After drying the sections, primary antibodies: anti-ALP (1:200, Huabio, China), anti-COL1A1 (1:200, Cell Signaling Technology, USA), anti-OSX (1:200, Abcam, UK), and anti-OCN (1:200, Huabio, China) were added, each diluted according to the recommended ratio, and the samples were incubated overnight at 4°C. After 1 h RT equilibration, sections were incubated with fluorescent secondary antibody (Solarbio, China) in darkness for 1 h, washed 3 times with PBST, counterstained with DAPI (Solarbio, China) for 30 min, and mounted.

QUANTIFICATION AND STATISTICAL ANALYSIS
Statistical analyses were performed using GraphPad Prism software. Data are presented as mean ± standard deviation (SD) from at least three independent experiments. Comparisons between two groups were analyzed using Student's *t* test, while multiple group comparisons were analyzed using one-way ANOVA followed by Tukey's post-hoc test. Statistical significance was defined as $p < 0.05$. Sample sizes (n) for each experiment are indicated in the figure legends.

Stem Cell Reports, Volume *21*

Supplemental Information

HDAC4 regulates hASC osteogenesis and bone regeneration by mediating SMAD4 via histone acetylation

Liyuan Yu, Kai Xia, Yijue Wang, Zhiai Hu, Jun Liu, Shujuan Zou, and Jianwei Chen

Supplemental Information

Document S1. Figures S1–S6

Table S1. Synthesized sequences of siRNA.

Table S2. Primer sequences of RT-qPCR.

Table S3. Primer sequences of CUT&Tag-qPCR

Table S4. Primer sequences of Human-specific Alu

1. Figure S1 CUT&Tag sequencing revealed HDAC4 inhibition enhances H3 acetylation modification during hASC osteogenic differentiation.

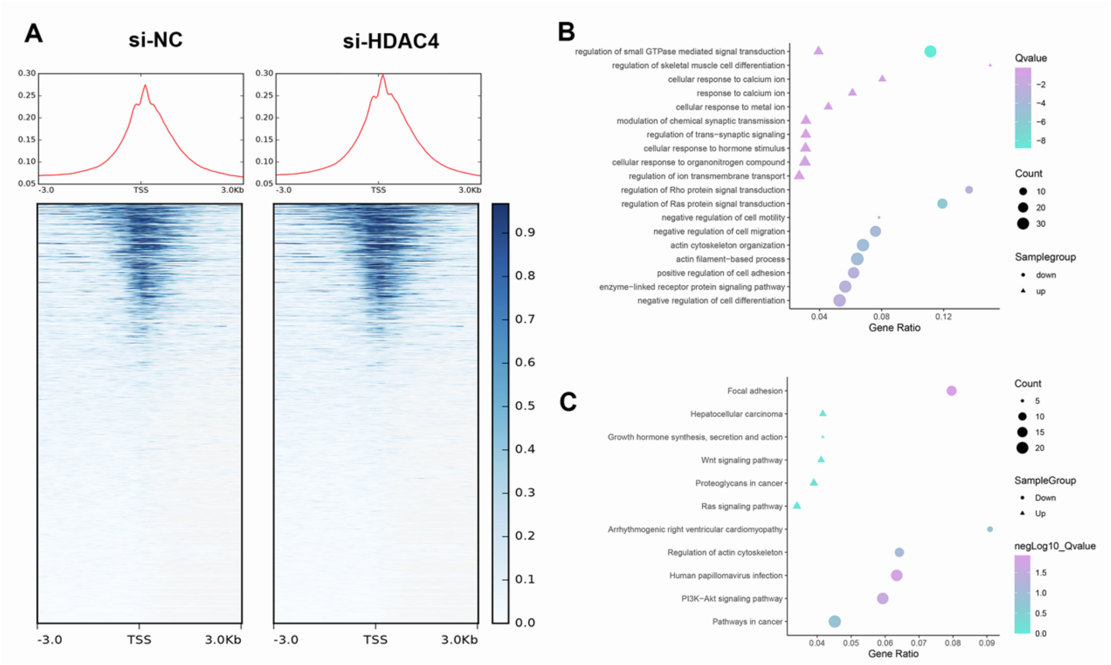

(A) The line graph shows the peaks identified by CUT&Tag enrichment around the transcription start site (TSS). The x-axis represents the distance from TSS, while the y-axis represents the degree of peak enrichment. The clustered heatmap reveals enriched peaks near the TSS, with blue indicating the level of enrichment. (B) GO analysis of differential peaks. (C) KEGG analysis of differential peaks.

2. Figure S2 Knockdown of HDAC4 promotes cranial bone defect repair.

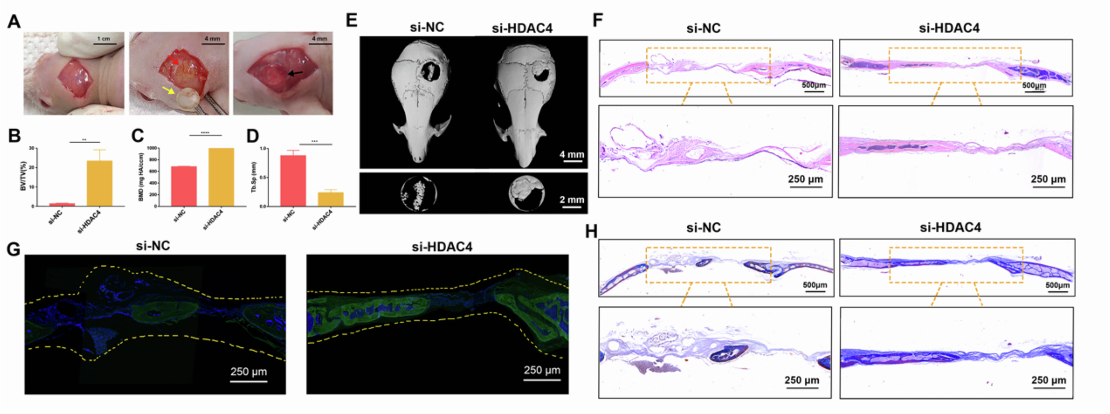

(A) Surgical images show the establishment of a critical-sized calvarial defect model in mice and the implantation of hydrogel microspheres. Red arrow indicates the defect area; black arrow indicates the implanted microspheres; yellow arrow indicates the explanted calvarial tissue. Scale bars: 1 cm (overview), 4 mm (magnified view). (B, C, D) Quantitative micro-CT analysis of bone repair parameters: bone volume fraction (BV/TV), bone mineral density (BMD) and trabecular spacing (Tb.Sp) in si-HDAC4 versus si-NC groups. Data are mean ± SD (n = 5 mice per group from three independent experiments). Statistical significance was determined by an unpaired two-tailed t-test. $^{**}p < 0.01$, $^{***}p < 0.001$, $^{****}p < 0.0001$. (E) The three-dimensional reconstruction of the cranial bone of si-HDAC4 and si-NC groups. Lower images show magnified views of the defect areas. Scale bars: 4 mm (overview), 2 mm (magnified view). (F) Hematoxylin and eosin staining. Scale bars: 500 μm (overview), 250 μm (magnified view). (G)The immunofluorescence staining of the osteogenic marker COL1A1 (green); nuclei were counterstained with DAPI (blue). Scale bar: 250 μm. (H) Masson staining with blue staining indicating collagenous matrix. Scale bars: 500 μm (overview), 250 μm (magnified view).

3. Figure S3 High-resolution histological analysis of mandibular defect repair delineating the interface between pre-existing host bone and new bone formation.

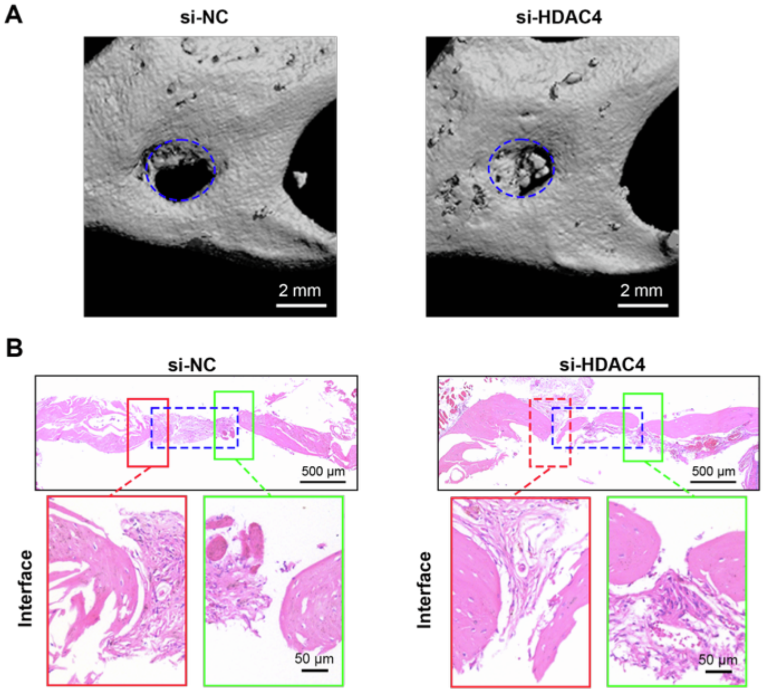

(A) Micro-CT 3D reconstruction of the mandibular defect region. The blue dashed circle indicates the central area of the original critical-sized defect. Scale bar: 2 mm. (B) Hematoxylin and eosin staining show the overall defect repair area. The blue dashed box outlines the central area of the original critical-sized defect. The red and green boxes indicate high-magnification views of the interface: the pre-existing host bone at the defect margin, characterized by dense, lamellar architecture; and the newly formed bone within the defect, displaying immature, porous woven bone. Scale bars: 500 μm (overview), 50 μm (insets).

4. Figure S4 SMAD4 knockdown inhibits osteogenic differentiation of hASCs.

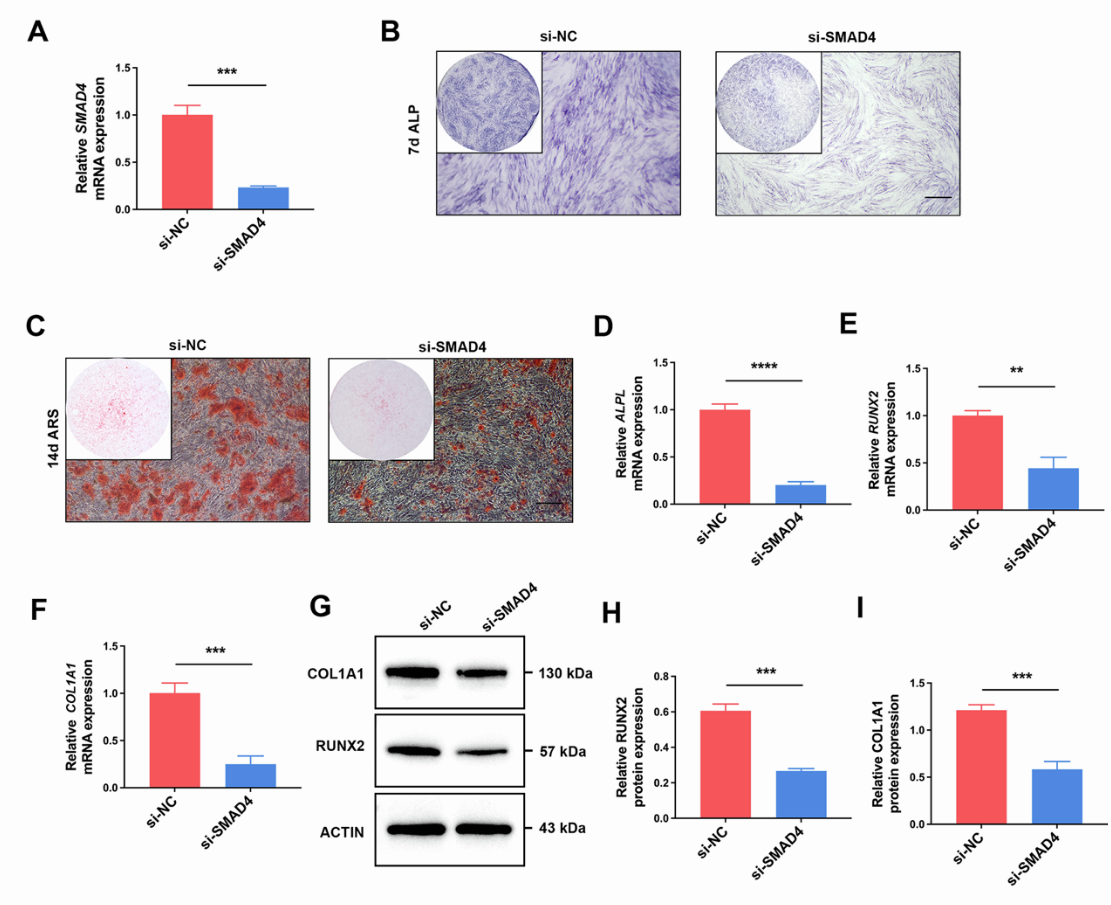

(A) RT qPCR analysis of *SMAD4* mRNA expression in hASCs following transfection with si-SMAD4 or si-NC. Data are mean ± SD (n = 3 independent experiments). Statistical significance was determined by an unpaired two-tailed t-test. ***$p < 0.001$. (B, C) ALP staining at day 7 and ARS staining at day 14 under osteogenic induction in si-SMAD4 versus si-NC. Scale bars: 200 μm. (D, E, F) RT-qPCR analysis of the osteogenic marker genes *ALPL*, *RUNX2*, and *COL1A* in si-SMAD4 versus si-NC. Data are mean ± SD (n = 3 independent experiments). Statistical significance was determined by an unpaired two-tailed t-test. **$p < 0.01$, ***$p < 0.001$, ****$p < 0.0001$. (G) Western blot detects the protein expression of RUNX2 and COL1A1 in si-SMAD4 versus si-NC. (H, I) Quantification of the protein levels of RUNX2 and COL1A1. Data are mean ± SD (n = 3 independent experiments). Statistical significance was determined by an unpaired two-tailed t-test. ***$p < 0.001$.

5. Figure S5 Composition Schematic Diagram of GelMA-F127 Hydrogel Loaded with Tasquinimod.

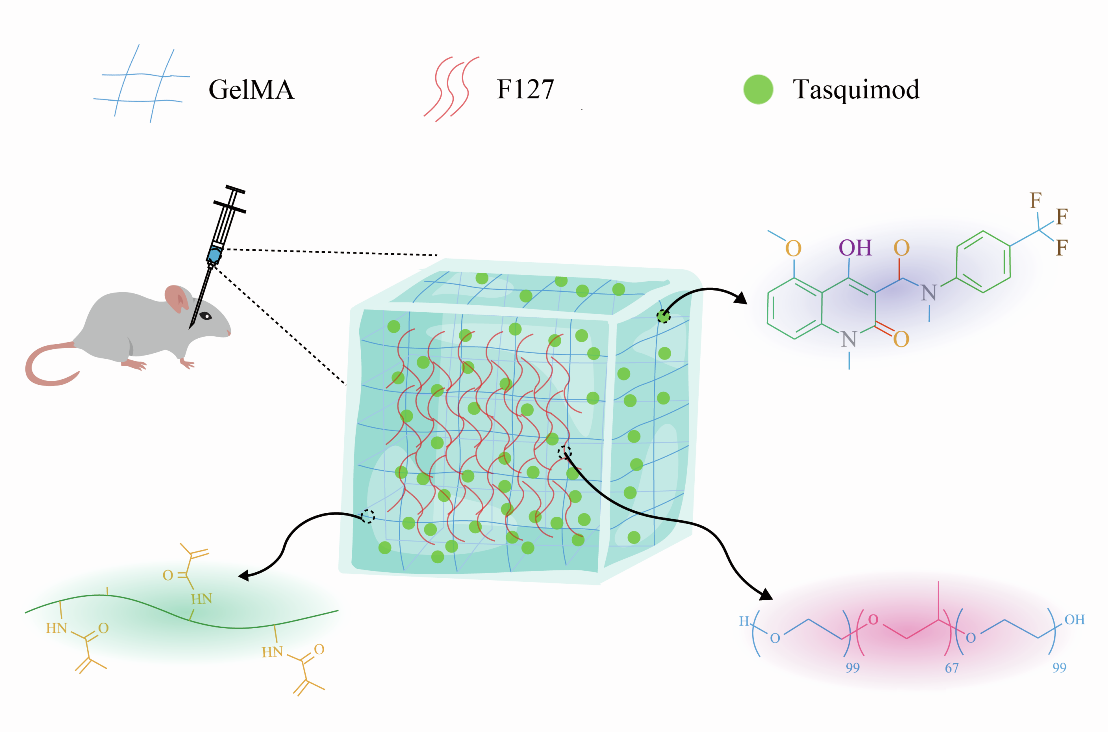

6. Figure S6 *In vitro* Tasqunimod release kinetics and *in vivo* engraftment verification of hASCs.

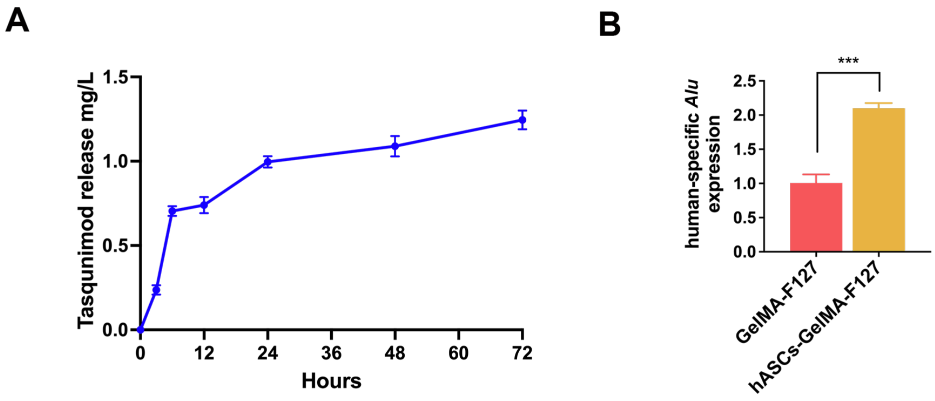

(A) *In vitro* release profile of Tasquinimod (Tasq) from GelMA-F127 hydrogel over 72 hours in PBS (pH 7.4, 37°C). Data are presented as mean ± SD (n = 3 independent experiments). The profile shows an initial rapid release phase followed by sustained release. (B) In vivo detection of transplanted human cells. Genomic DNA from mandibular defect sites 8 weeks post-implantation was analyzed by qPCR for human-specific *Alu* sequences. A significant signal was detected in the hASCs-GelMA-F127 group compared to the cell-free control (GelMA-F127), confirming donor cell engraftment. Data are presented as mean ± SD (n = 3 independent experiments). Statistical significance was determined by an unpaired two-tailed Student's t-test (***p < 0.001).

Table S1. Synthesized sequences of siRNA.

Genes	Sequences (5'-3')
HDAC4	GCAGCAGCAUCAGCAGUUUTT
	AAACUGCUGAUGGUGCUGCTT
SMDA4	GGUGGAGAGAGUGAAACAUTT
	AUGUUUCACUCUCUCCACCTT
NC	UUCUCCGAACGUGUCACGUTT
	ACGUGACACGUUCGGAGAATT

Table S2. Primer sequences of RT-qPCR.

Genes	Primer sequences (5'-3')
**GAPDH**	Forward: CTTTGGTATCGTGGAAGGACTC<br>Reverse: GTAGAGGCAGGGATGATGTTCT
**SMAD4**	Forward: ACTCCTACAACCTAGCCACCTTCTC<br>Reverse: GTTCTGTGTTGAGGACCTGCGTAG
**BGLAP**	Forward: CTACCTGTATCAATGGCTGGG<br>Reverse: GGATTGAGCTCACACACCT
**HDAC4**	Forward: GCCAAAGATGACTTCCCTCTTA<br>Reverse: TTTCGGCCACTTTCTGCTTTAG
**COL1A1**	Forward: CCAGTGTGGCCCAGAAGAAC<br>Reverse: TGGCCGCCATACTCGAACT
**MAP2K2**	Forward:CCTACATGGCTCCACCTCCTAAG<br>Reverse:ACCTCGGACCGCTTGATGAAG
**AKT2**	Forward:TGCGGAAGGAAGTCATCATTGC<br>Reverse:TCGTGGGTCTGGAAGGCATAC
**HDAC7**	Forward:ACCTCAATGCCATCCGCTCTC<br>Reverse:CACTGCCTCCACTTCTTCTTTGTC
**HDAC5**	Forward:ACGCTAGATGAGATCCAGACAGTG<br>Reverse:CCACAAGGCAGCACAGCATAC

Table S3. Primer sequences of CUT&Tag-qPCR

Genes	Primer sequences (5'-3')
**SMAD4**	Forward: CACGCACAGTCCTTGGTTGG
	Reverse: CTGATGCTGCTGCTGGATGAC

Table S4. Primer sequences of Human-specific Alu

Genes	Primer sequences (5'-3')
	Forward: TCGCCCAGGCTGGAGTGCA
	Reverse: CACCTGTAATCCCAGCACTTT
